# Payments to Physician Practices and Incentives to Serve Different Racial and Ethnic Groups

**DOI:** 10.1001/jamahealthforum.2025.4561

**Published:** 2025-11-26

**Authors:** Aaron L. Schwartz, David A. Asch, Rachel M. Werner

**Affiliations:** 1 Department of Medical Ethics and Health Policy, Perelman School of Medicine, University of Pennsylvania, Philadelphia; 2 Division of General Internal Medicine, Department of Medicine, Perelman School of Medicine, University of Pennsylvania, Philadelphia; 3 Leonard Davis Institute of Health Economics, University of Pennsylvania, Philadelphia; 4 Corporal Michael J. Crescenz VA Medical Center, Philadelphia, Pennsylvania; 5 Agency for Healthcare Research and Quality, Rockville, Maryland

## Abstract

**Importance:**

In the US, a physician can be paid very different amounts for treating different patients, even when providing identical services. Understanding physician practices’ financial incentives to serve different racial and ethnic groups may help inform payment policies to reduce health disparities.

**Objective:**

To measure disparities across patient racial and ethnic groups in per-visit payment to physician practices from health insurers and other sources, adjusted for visit content, geographic market, and year, and to quantify the role of health insurance source and other factors in these disparities.

**Design, Setting, and Participants:**

A unique, nationally representative dataset of outpatient visits containing survey-obtained patient race and ethnicity and payment amounts to physician practices from health insurers and other sources was analyzed. Data were collected from 2014 to 2021.

**Main Outcomes and Measures:**

Payment disparities were defined as gaps between patient groups defined by race and ethnicity in total payments per visit to physician practices, adjusted for visit content, geographic market, and year. Kitagawa-Oaxaca-Blinder decompositions were used to estimate the magnitude of these disparities and to quantify the roles of factors like health insurance.

**Results:**

The sample included 38 722 patients and 152 336 outpatient visits for evaluation and management services; a total of 8126 (21.0%) were Hispanic, 6150 (15.9%) were non-Hispanic Black, and 24 446 (63.1%) were non-Hispanic White. A total of 152 336 outpatient visits were included for evaluation and management services. In adjusted analyses, outpatient payments were 8.8% (95% CI, 6.7-11.0) less for visits with non-Hispanic Black patients and 9.8% (95% CI, 7.2-12.4) less for visits with Hispanic patients compared with visits with non-Hispanic White patients. Payment gaps were largest for children (13.9% [95% CI, 11.8-16.0] for non-Hispanic Black children; 15.1% [95% CI, 12.8-17.4] for Hispanic children), smaller when adjusted for insurance source (4.9% [95% CI, 2.7-7.1] for non-Hispanic Black patients; 5.6% [95% CI, 3.0-8.3] for Hispanic patients), and absent among patients with fee-for-service Medicare (1.2% [95% CI, −1.5 to 3.9] for non-Hispanic Black patients; −0.6% [95% CI, −4.4 to 3.2] for Hispanic patients).

**Conclusions and Relevance:**

In this study, US physician practices were paid more for outpatient visits with non-Hispanic White patients than for outpatient visits with Hispanic or non-Hispanic Black patients. Payment disparities were larger in pediatrics and partly explained by insurance. Differential financial incentives to serve non-Hispanic White patients may worsen disparities in health care access, utilization, and quality.

## Key Points

**Question:**

Are payments to US physician practices larger for outpatient visits with non-Hispanic White patients than for similar visits with Hispanic or non-Hispanic Black patients?

**Findings:**

In this nationally representative sample of 152 336 outpatient visits from 2014 to 2021, total payments to physician practices from health insurers and other sources were 8.8% less for non-Hispanic Black patients and 9.8% less for Hispanic patients compared with non-Hispanic White patients. These gaps, which were adjusted for visit content, geographic market, and year, were largest for pediatric visits (13.9% for non-Hispanic Black children and 15.1% for Hispanic children) and smaller when also adjusted for insurance source (4.9% and 5.6%, respectively).

**Meaning:**

Differential payments to physician practices by patient race and ethnicity may worsen disparities in health care access, utilization, and quality.

## Teaser

This study measures disparities across patient racial and ethnic groups in per-visit payment to physician practices from health insurers and other sources, adjusted for visit content, geographic market, and year, and quantifies the role of health insurance source and other factors in these disparities.

## Introduction

In the US, a physician can be paid very different amounts for providing the same service to different patients. Such differences arise within a patchwork system of health care financing that distinguishes the US from similar nations.^1^ For example, Medicaid professional fees are about 28% less than Medicare payments, which are 29% less than commercial insurance fees.^2,3^ This variation in payment creates incentives to treat patients enrolled in higher-paying insurance plans and to turn away patients enrolled in lower-paying plans.^4^ Patients in racial and ethnic minority groups, who are disproportionately enrolled in lower-paying insurance plans, may be more likely to face such barriers to health care access.^5,6,7^

Measuring differential payments to physician practices across patient groups defined by race and ethnicity can illustrate the differential incentives and resources for serving these patient groups. Quantifying these payment gaps can also highlight the role of payment policies in contributing to health disparities and could inform payment reform efforts. Increasing payments to physicians from insurers can increase health care access,^8,9^ utilization,^10,11,12,13^ and quality.^10,14,15,16^ Therefore, the well-documented racial and ethnic disparities in these domains^17^ may arise partly from gaps in payment generosity. While health disparities arise from disparate proximal causes, including implicit and explicit bias (eg, a clinician unfairly treating patients differently)^18,19,20,21^ and structural inequalities (eg, lower-quality facilities serving patients of racial and ethnic minority groups),^22^ gaps in payment may be a shared, addressable contributor to these patient harms (see eAppendix in Supplement 1).

We sought to measure race- and ethnicity-associated differences in physician practice payments and to quantify the magnitude of any payment reduction associated with treating patients in racial and ethnic minority groups, specifically Black and Hispanic patients. Researchers have often used statistical methods to measure health care disparities by estimating differences in outcomes across groups, adjusted for relevant differences in group characteristics.^23^ However, data limitations have been a key obstacle to extending such methods to the topic of provider payment. Prior research has been limited by available data, which often lacks some of the elements necessary to measure payment disparities, including encounter-level payments, patient demographic characteristics, details on services provided, and geographic location, for a large and nationally representative sample of outpatient visits spanning insurance types. We overcome this obstacle by using a unique data source with all these features, allowing us to estimate the average total visit payment to outpatient clinical practices from all sources, by patient race and ethnicity, adjusted for visit content, geographic market, and year.

## Methods

### Data and Study Sample

We conducted a cross-sectional analysis of outpatient visits from the 2014 to 2021 Medical Expenditure Panel Survey (MEPS), a nationally representative survey of the civilian noninstitutionalized population in the US. Typical household respondents are interviewed in-person for 5 rounds covering 2 calendar years and provide detailed information on health care use, health status (eg, respondent-reported health), insurance, and various characteristics of household members. The MEPS Medical Provider Component gathers additional billing information about health care encounters. MEPS Medical Provider Component data are derived from telephone surveys conducted with a sample of physician practices where MEPS households received care. For each surveyed visit, practices’ billing offices provide all *Current Procedural Terminology, fourth edition*/Healthcare Common Procedure Coding System (HCPCS) procedure codes and charges, total charges, and all payments made to the practice by payment source. Because of these detailed data elements, MEPS data are widely used for tracking not only health care use but also health care expenditures and prices. This study was approved by the Advarra Institutional Review Board governing Agency for Healthcare Research and Quality analyses of MEPS microdata, and informed consent was waived due to minimal participant risk. We followed the Strengthening the Reporting of Observational Studies in Epidemiology (STROBE) reporting guideline.

To supplement these MEPS data, we used additional data describing service and market characteristics. For each procedure code and each year, we obtained total relative value units (RVUs) from the Centers for Medicare & Medicaid Services RVU files and average payments for commercial insurers in MarketScan. For each household zip code in each year, we obtained Centers for Medicare & Medicaid Services Geographic Practice Cost Index adjustment factors from the RVU files, physician practice concentration measures derived from 5% samples of Medicare fee-for-service (FFS) claims^24^ and sociodemographic statistics from the Agency for Healthcare Research and Quality Social Determinants of Health Database (eMethods in Supplement 1).

Our initial sample included all household-reported outpatient visits with matching billing data. We excluded visits in hospital outpatient departments because of data incompleteness and complexities involved with facility billing components (eMethods in Supplement 1). We also excluded visits in emergency departments, in facilities within the military, Veterans Health Administration, or Indian Health Services and visits in which procedure codes were entirely absent from the Medicare RVU file and MarketScan outpatient file. We excluded visits lacking procedure codes for evaluation and management services (CPT-4/HCPCS codes 99201-99215, 99381-99429, and G0438-G0439) and visits with outlier total charges above the 99th percentile. Finally, due to sample size limitations for other racial and ethnic groups, we restricted our sample to individuals identified by household survey respondents as Hispanic, non-Hispanic Black, or non-Hispanic White (eTable 1 in Supplement 1). Although sample size limitations precluded analysis of race and ethnicity separately, in a sensitivity analysis, we redefined mutually exclusive race and ethnicity categories as Black, non-Black Hispanic, and non-Hispanic White.

### Defining Payment Disparities

To define and measure payment disparities, we adapted established methods for defining and measuring disparities in health care utilization.^23^ Our primary operational definition of payment disparities isolates differences in payment that would occur if a physician were to provide the same visit content, in the same location and year, to patients from different racial and ethnic groups. Thus, in our primary analysis, we adjusted payment gaps for differences in visit characteristics, location and market characteristics, and year. In analyses of systemic sources of disparities, selecting these adjustment factors is a key methodological decision; adjustment for too many factors can introduce statistical bias.^25^ Notably, our primary estimates are not adjusted for differences in insurance characteristics, sociodemographic characteristics, or health status. In secondary analyses, we measured unadjusted payment gaps and payment gaps adjusted for a different set of factors (eg, with additional adjustment for insurance characteristics). See the eMethods in Supplement 1 for a detailed discussion of defining payment disparities.

### Variables for Disparity Estimation

For each outpatient visit, we calculated total payments to the practice from all sources, including insurers and out-of-pocket payments, inflation-adjusted to 2021 dollars using the National Health Expenditure Accounts physician price index. We accounted for differences in visit characteristics using total visit RVUs and RVUs-squared, indicators for household-reported visit purpose (eg, general checkup, immunizations), indicators for provider type (physician primary care, physician specialist, or nonphysician clinician), indicators for physician specialty (a 3-tier measure based on specialty-specific payment amounts), and indicators for any associated laboratory or radiologic testing. For CPT-4/HCPCS codes without matching RVUs (3.1% of all charges), we measured the intensity of these services as the line charges for the codes. We accounted for differences in location and market using indicators for 266 hospital referral regions (HRRs) and zip code–level Herfindahl-Hirschman Indices of primary care concentration and multispecialty practice concentration. Because our data contained a small sample size of visits within each physician practice, it was not feasible to compare payment rates across patient race and ethnicity within each practice, which would have accounted for additional practice-level characteristics. However, in a sensitivity analysis, we incorporated additional practice characteristics, including provider count, location count, specialty mix, and health system affiliation, using a subsample of practices that participated in the MEPS Medical Organizations Survey (n = 29 943 visits).

Following established methods to address confounding in disparity estimation,^23,26^ our statistical models included additional variables that may be correlated with visit content, geographic market, and year. These additional variables contained insurance characteristics, sociodemographic characteristics, and health status details. Insurance was defined according to a 7-category measure of household-reported health insurance coverage. Sociodemographic characteristics included patient age, gender, marital status, educational attainment, income relative to the federal poverty level, education, county-level racial and ethnic composition, and racial and ethnic category (Hispanic, non-Hispanic Black, and non-Hispanic White). Health status was defined using household-reported health ratings, activity limitations, chronic conditions, hospitalizations, home health use, and death/institutionalization during the survey year. See eTables 2 and 3 in Supplement 1 for comprehensive descriptions of all variables used in our estimation.

### Statistical Analysis

To estimate payment disparities, ie, adjusted differences in payment to physician practices across patient racial and ethnic groups, we conducted Kitagawa-Oaxaca-Blinder decompositions using pooled, linear regression modeling. A detailed description of this approach is in the eMethods in Supplement 1. Briefly, we estimated a linear regression model of total visit payment as a function of the variables described above: year, visit, and market characteristics, as well as insurance, sociodemographic and health characteristics (eMethods in Supplement 1). We used the estimated coefficients from this model to calculate the average expected payment per visit for each of the 3 racial and ethnic groups (Hispanic, non-Hispanic Black, and non-Hispanic White patients); these expected payment amounts were adjusted as if visits across every racial and ethnic group had the same values for year indicators, visit characteristics, and market characteristics as the mean for visits with non-Hispanic White patients (eMethods in Supplement 1). These average expected payments were used to calculate our primary outcomes: (1) the percentage difference in adjusted payment between visits with non-Hispanic Black patients and visits with non-Hispanic White patients and (2) the percentage difference in adjusted payment between visits with Hispanic patients and visits with non-Hispanic White patients. Confidence intervals for these payment disparities were estimated via the delta method. To estimate how much the elimination of these payment disparities might reduce disparities in health care use, we conducted an exploratory simulation based on published estimates of how clinician payment levels affect health care utilization (eMethods in Supplement 1).

We quantified payment disparities for visit subgroups defined by patient, visit, or market characteristics, testing for subgroup differences via Wald tests. To explore the role of health insurance in payment disparities, we conducted 3 additional analyses. First, in a falsification test of our research design, we estimated payment disparities within a subsample of patients with FFS Medicare. Because FFS Medicare standardizes physician payment according to visit content, location, and year, no payment disparities should exist in this subsample. Detecting payment disparities in this subsample could suggest systemic errors in key variables or model misspecification. Second, to test whether payment disparities exist for patients with similar health insurance, we conducted subgroup analyses by health insurance source. Third, we conducted a decomposition analysis, recalculating payment gap estimates adjusted for differences across racial and ethnic groups in insurance source; we also recalculated payment gap estimates adjusted for differences across racial and ethnic groups in demographic, health, and socioeconomic characteristics besides racial and ethnic identity. Comparing our primary estimates to these alternate estimates quantifies the extent to which payment disparities are explained by differences in insurance status or by other differences in patient characteristics. Finally, we conducted various sensitivity analyses with modifications to our sample, variable definitions, or statistical model (eMethods in Supplement 1).

Significance was set at *P* < .05, and all *P* values were 2-sided. All analyses were conducted using STATA/MP version 18 (StataCorp) and corrected for the complex multistage clustered and stratified design of the MEPS (eMethods in Supplement 1).

## Results

### Sample Characteristics

**Figure 1.**
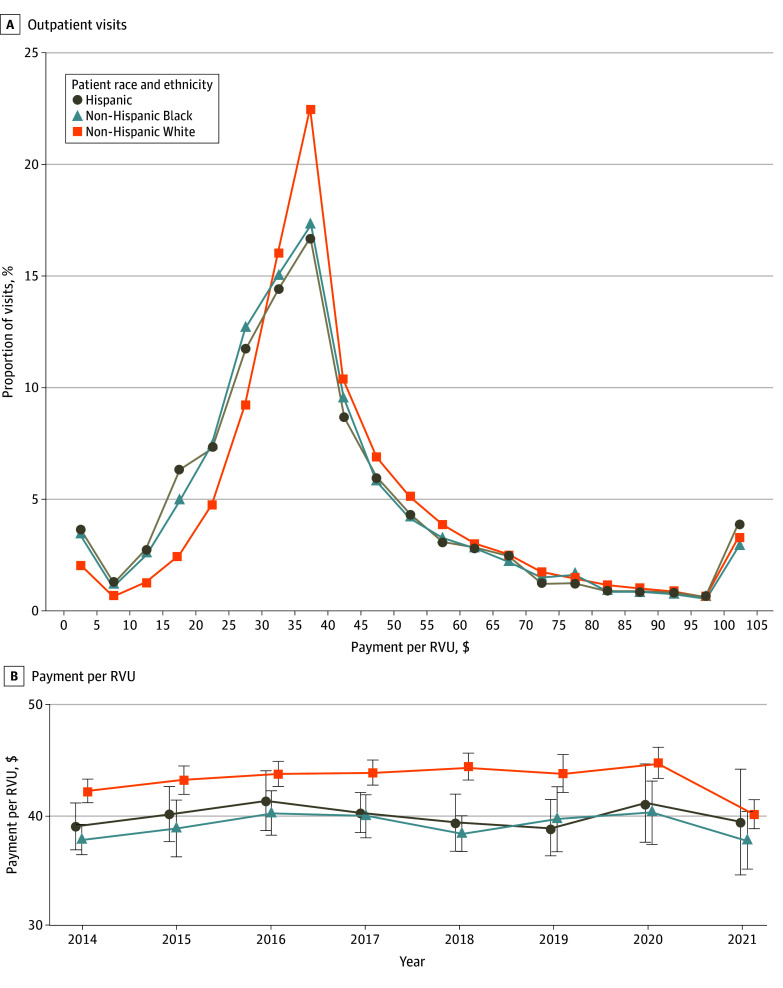
Payments for Outpatient Visits by Patient Racial and Ethnic Group A, Distribution across visits in unadjusted total payments per total relative value units (RVUs). Total payments refer to payments to physician practices from all sources including insurer and out-of-pocket payments. Total RVUs include all RVU components (ie, work, facility, and malpractice). Frequency is calculated within bins that are $5/RVU wide, with payment winsorized (top-coded) at $105/RVU for this visualization. B, Unadjusted total payment per RVU over time. Racial and ethnic categories are mutually exclusive and were obtained via household phone survey. All results are weighted to account for complex survey sampling. Error bars indicate 95% CIs.

Our sample of 152 336 outpatient visits included 38 772 unique patients, of whom 8126 (21.0%) were Hispanic, 6150 (15.9%) were non-Hispanic Black, and 24 446 (63.1%) were non-Hispanic White (Table 1). Health insurance sources differed between these groups; Medicaid was the sole insurance source for 30.0%, 23.2%, and 9.1% of Hispanic, non-Hispanic Black, and non-Hispanic White patients’ visits, respectively. Visit intensity was similar across groups according to mean (SE) total RVUs (3.29 [0.03], 3.30 [0.03], and 3.38 [0.01], respectively). Mean (SE) unadjusted payments for visits with White patients were $146 (1.1) per visit compared with $131 (2.3) for visits with Hispanic patients (10.2% less) and $129 (1.8) for visits with Black patients (11.8% less). Payments per RVU were highest for visits with White patients in every year of the study period (Figure 1). eTable 4 in Supplement 1 includes extended descriptive statistics.

**Table 1.** Outpatient Visits by Patient Racial and Ethnic Group^a^

Characteristic	%		
	Hispanic	Non-Hispanic Black	Non-Hispanic White
Encounters, No.	27 115	22 036	103 185
Unique patients, No.	8126	6150	24 446
Payment from all sources			
Payment per visit, mean (SE), $	131 (2.3)	129 (1.8)	146 (1.1)
Payment per work RVU, mean, $	40	39	43
Patient sociodemographic and health characteristics			
Age, y			
0-17	29.8	15.4	13.9
18-64	53.1	60.7	51.3
≥65	17.2	23.9	34.8
Gender			
Female	57.7	64.4	58.1
Male	42.3	35.6	41.9
No college degree in family	65.4	65.7	47.3
Family income below FPL	20.5	22.9	8.7
Self-rated health as excellent or very good	48.5	41.9	52.5
Count of health conditions	1.5	2.2	2.1
Any ADL limitation	13.2	21.5	14.7
Visit characteristics			
Primary care physician	57.9	52.1	43.5
Specialty physician	30.3	34.0	37.9
Nonphysician clinician	11.7	13.9	18.6
Work RVUs, mean (SE)	3.29 (0.03)	3.30 (0.03)	3.38 (0.01)
Insurance characteristics			
Any Medicare	21.9	34.1	39.5
Private source without Medicare	42.8	39.2	49.1
Medicaid only	30.0	23.2	9.1
Uninsured	5.3	3.5	2.4
Market characteristics			
Northeast	18.2	14.9	20.4
Midwest	10.1	15.9	22.6
South	42.5	64.1	39.3
West	29.2	5.1	17.6

Abbreviations: ADL, activity of daily living; FPL, federal poverty level; RVU, relative value unit.

^a^ This table contains a limited set of descriptive characteristics and several categorical variables that were pooled for brevity (eg, age). Comprehensive summary statistics of all variables included in our analyses are presented in eTables 2 to 4 in Supplement 1. All results are weighted to account for complex survey sampling.

### Payment Disparity Estimates

**Figure 2.**
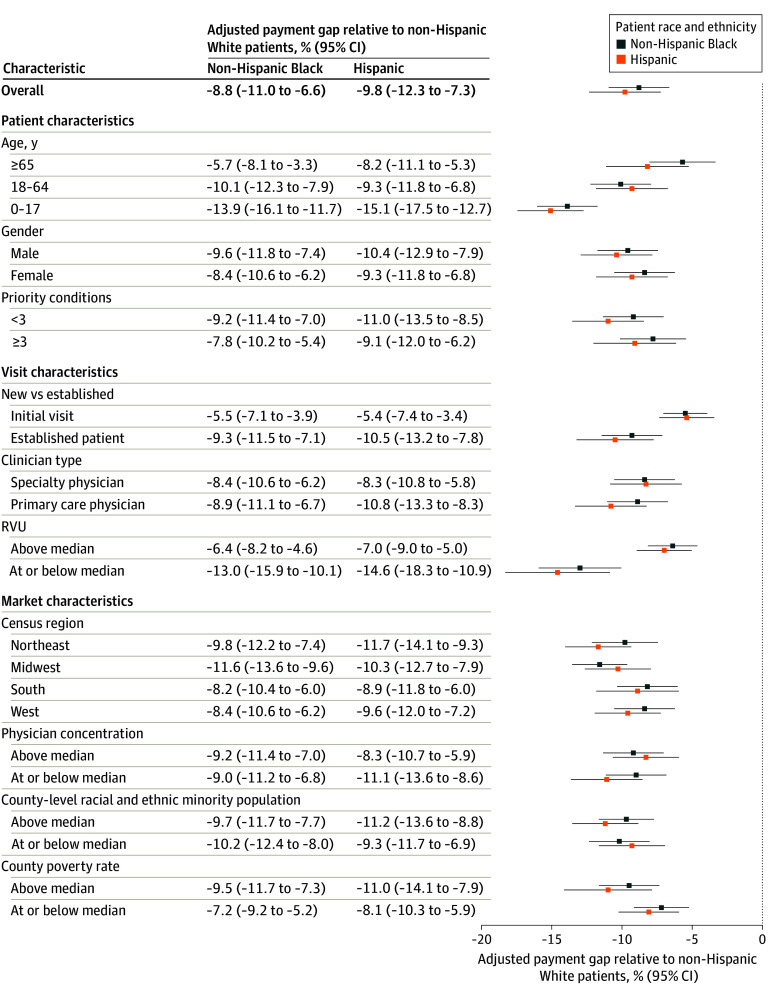
Payment Disparities for Outpatient Visits Compared With Non-Hispanic White Patients by Subgroup Payment gaps are adjusted for differences in visit characteristics, market characteristics, and year of visit. See eMethods in Supplement 1 for regression specification and other details of statistical analyses. Priority conditions include coronary heart disease, angina, myocardial infarction, other unspecified heart disease, stroke, emphysema, high cholesterol, cancer, arthritis, diabetes, asthma, joint pain, and chronic bronchitis. All results are weighted to account for complex survey sampling. RVU indicates relative value unit.

The payment received by physician practices was 8.8% (95% CI, 6.7-11.0; *P* < .001) less for visits with non-Hispanic Black patients and 9.8% (95% CI, 7.2-12.4; *P* < .001) less for visits with Hispanic patients than for visits with non-Hispanic White patients, after adjustment for visit content, geographic market, and year. Payment disparities were present within every subgroup of visit categories, though the magnitude of disparities varied (Figure 2). The greatest disparities were present for patients aged 0 to 17 years; relative to similar visits with White children, payments were 13.9% (95% CI, 11.8-16.0) less for visits with Black children and 15.1% (95% CI, 12.8-17.4) less for visits with Hispanic children. Among visits with adults 65 years and older, payment disparities were smaller, with an estimated 5.7% (95% CI, 3.4-7.9) gap between Black and White patients and an 8.2% (95% CI, 5.2-11.2) gap between Hispanic and White patients. Simulations suggest that eliminating payment disparities of these magnitudes could substantially narrow or eliminate racial and ethnic disparities in health care use (eTable 7 and the eFigure in Supplement 1).

In subgroup analyses (Table 2; eTable 5 in Supplement 1), Wald tests indicated statistically significant differences in payment disparities for most comparisons across visit categories defined by patient, visit, or market characteristics. However, payment disparities were not statistically different in markets with greater vs lesser physician concentration (an indicator of market power in price negotiations) or in markets with greater vs lesser minority population share. In counties with poverty rates above the median, payment disparities between Black and White patients were 2.4 percentage points (−9.5% vs −7.2%, respectively) larger and disparities between Hispanic and White patients were 2.9 percentage points (−11.0% vs −8.1%, respectively) larger.

**Table 2.** Falsification Tests, Subgroup Analysis, and Decomposition of Payment Gaps Based on Insurance Source

Measure	Payment gap (95% CI), %	
	Difference between Black and White patients	Difference between Hispanic and White patients
**Falsification test^a^**		
Subgroup		
Fee-for-service Medicare	−1.2 (−3.9 to 1.5)	0.6 (−3.2 to 4.4)
Fee-for-service Medicare, new patients only	0.1 (−5.2 to 5.3)	−1.5 (−8.9 to 5.8)
**Subgroup analysis^a^**		
Insurance status^b^		
Any Medicare	−5.4 (−7.8 to −3.0)	−6.1 (−9.1 to −3.1)
Private source without Medicare	−3.9 (−5.8 to −2.0)	−4.4 (−6.7 to −2.1)
Medicaid only	−5.0 (−8.1 to −1.9)	−5.6 (−9.6 to −1.6)
Uninsured	−5.1 (−7.6 to −2.7)	−5.8 (−8.8 to −2.9)
**Decomposition**		
Adjustments for payment differences		
Unadjusted	−11.8 (−13.6 to −10.1)	−10.2 (−12.0 to −8.4)
Year, visit, and market^c^	−8.8 (−11.0 to −6.7)	−9.8 (−12.4 to −7.2)
Year, visit, market, and insurance	−4.9 (−7.1 to −2.7)	−5.6 (−8.3 to −3.0)
Year, visit, market, sociodemographic, and health	−8.0 (−10.2 to −5.8)	−8.5 (−11.2 to −5.9)

^a^ Comparisons are adjusted for allowable differences in visit characteristics, market characteristics, and year of visit. See eMethods in Supplement 1 for regression specification and other details of statistical analyses.

^b^ Categories of insurance status are mutually exclusive.

^c^ Estimates correspond to overall payment gaps presented in Figure 2.

### Role of Health Insurance

Table 2 demonstrates the role of patients’ health insurance in the magnitude of outpatient payment disparities across patient groups defined by race and ethnicity. There were no payment disparities for visits with Black and Hispanic patients relative to White patients among those with FFS Medicare, which standardizes payment according to visit content, geographic market, and year. However, there were payment disparities for patients within broader categories of insurance, which do not similarly standardize physician payment. Among the uninsured, payments for visits with Black patients were 5.1% (95% CI, −7.6 to −2.7) less than for similar visits with White patients and 5.8% (95% CI, −8.8 to −2.9) less for visits with Hispanic patients than for similar visits with White patients. For every insurance status, visits with patients sharing that insurance status had smaller racial and ethnic payment gaps than the corresponding payment gaps for the overall sample.

In decomposition analyses (Table 2; eTable 6 in Supplement 1), insurance differences across racial and ethnic groups were a key factor in payment disparities. Compared with unadjusted payment gaps, estimated payment gaps were modestly smaller after adjustment for differences in visit content, geographic market, and year. However, estimated payment gaps narrowed substantially (to a difference of 4.9% [95% CI, −7.1 to −2.7] between Black and White patients and a difference of 5.6% [95% CI, −8.3 to −3.0] between Hispanic and White patients) after adjustment for insurance status (ie, with insurance coverage equalized across racial and ethnic groups). In contrast, payment gaps narrowed only modestly (to 8.0% [95% CI, −10.2 to −5.8] and 8.5% [95% CI, −11.2 to −5.9], respectively) if adjusted for sociodemographic and health differences.

### Sensitivity Analyses

In 25 of 28 sensitivity analyses, estimated outpatient payment disparities were as large or larger than the payment disparities calculated using our primary approach (Table 3). For example, payment disparities were 0.4 percentage points larger if collection costs were subtracted from total payments (difference between Black and White patients: −9.2% [95% CI, −11.3 to −7.1] vs −8.8 [95% CI, −11.0 to −6.7]; difference between Hispanic and White patients: −10.2% [95% CI, −12.8 to −7.6] vs −9.8% [95% CI, −12.4 to −7.2]),^27^ and were 0.4 to 3.0 percentage points larger when alternative definitions of service intensity were used. Redefining physician specialty categories, geographic regions, or insurance characteristics yielded modestly larger payment disparities. The only sensitivity analysis that yielded smaller payment disparities for both comparisons of Black with White patients and Hispanic with White patients was excluding visits for surgery and visits with Evaluation & Management G-codes, which reduced payment gaps by less than 0.2%.

**Table 3.** Sensitivity Analyses for Payment Disparity Estimation

Modification to primary estimate^a^	Payment gap (95% CI), %	
	Difference between Black and White patients	Difference between Hispanic and White patients
None^b^	−8.8 (−11.0 to −6.7)	−9.8 (−12.4 to −7.2)
Payment adjusted for collection costs	−9.2 (−11.3 to −7.1)	−10.2 (−12.8 to −7.6)
Services standardized with MarketScan price	−11.8 (−14.6 to −9.1)	−10.4 (−13.1 to −7.7)
Services standardized with work RVUs	−9.8 (−11.9 to −7.6)	−10.4 (−12.9 to −7.9)
RVUs modeled as piecewise spline	−9.3 (−11.4 to −7.1)	−10.7 (−13.3 to −8.1)
Narrow physician specialty categories used	−9.0 (−11.2 to −6.9)	−9.9 (−12.5 to −7.2)
MGPCI region used instead of HRR	−9.1 (−11.3 to −6.8)	−10.5 (−13.0 to −8.1)
Predictors of private insurance generosity added to insurance characteristics	−8.9 (−11.0 to −6.7)	−9.9 (−12.5 to −7.3)
Surgery visits and visits with Evaluation & Management G-codes excluded	−8.7 (−11.1 to −6.4)	−9.6 (−12.3 to −6.9)
Practice characteristics from MEPS Medical Organization Survey added to visit characteristics	−11.7 (−15.0 to −8.3)	−9.9 (−13.9 to −5.8)
Visits with charges above 99th percentile included	−8.0 (−12.6 to −3.4)	−10.9 (−14.2 to −7.6)
State Medicaid lesser-of policy included as insurance characteristic	−8.8 (−11.0 to −6.7)	−9.8 (−12.4 to −7.2)
Race and ethnicity categories redefined as Black, non-Black Hispanic, and non-Hispanic White	−8.8 (−11.0 to −6.7)	−9.8 (−12.4 to −7.2)
Payment modeled separately by race and ethnicity	−9.0 (−11.4 to −6.6)	−10.0 (−13.1 to −7.0)

Abbreviations: HRR, hospital referral region; MEPS, Medical Expenditure Panel Survey; MGPCI, Medicare Geographic Practice Cost Index; RVU, relative value unit.

^a^ All estimates of payment gaps are adjusted for visit characteristics, market characteristics, and year of visit. Definitions of visit or market characteristics may vary across sensitivity analyses. See eMethods in Supplement 1 for regression specification and other details of statistical analyses.

^b^ Estimates correspond to overall payment disparities presented in Figure 2.

## Discussion

In this observational study of outpatient clinic visits, we found that visits with Black or Hispanic patients yielded lower payments to physician organizations from insurers and other sources compared with visits with White patients. Payment gaps, which are largely explained by insurance source, were largest for visits with Black or Hispanic children, which yielded approximately 14% to 15% lower payments than similar visits with White children. We refer to these gaps as payment disparities because they reflect differential payment despite similar visit content (eg, RVUs), geographic market, and year. These payment disparities reflect financial incentives for physician practices to favor serving non-Hispanic White patients.

A key concern is that reduced fees for treating patients of racial and ethnic minority groups may contribute to downstream disparities in health care access and quality.^6,7^ Simple projections based on prior studies quantifying the effects of payment levels on health care utilization suggest that the payment disparities we measured could explain a substantial portion of known disparities in health care use (eTable 7 and the eFigure in Supplement 1).^28^ Still, the effect of payment disparities on patients is uncertain. Future studies could quantify the effects of payment disparities more directly by examining how racial and ethnic disparities in health care use and quality change following payment reforms. A second concern is that payment disparities are financially harmful to certain clinicians. There are large variations across physicians in the racial and ethnic mix of their patients^29^; future studies could quantify the effects of payment disparities on the well-being of clinicians who serve minority communities.

Our study contributes to prior research documenting differences in health care spending by race and ethnicity.^30,31,32^ These studies demonstrated greater spending for non-Hispanic White patients than Hispanic or non-Hispanic Black patients. Our analysis isolates the component of spending differences attributable to price differences, rather than differences in service utilization or geographic market. Measuring prices, ie, payments for the same services rather than spending per year or spending per visit, was possible because of the encounter-level details in our survey data, which included line-item procedure codes.

Differences in insurance coverage across racial and ethnic groups was one key contributor to the payment disparities we observed, accounting for 44% of the payment gap between Black and White patients and 43% of the payment gap between Hispanic and White patients. These findings highlight the role of public policies regarding payment generosity in producing payment disparities by patient race and ethnicity. In particular, narrowing the gap in payment generosity between Medicaid and other insurers would shrink racial and ethnic payment disparities.^33^ Although differences in source of insurance explain a large proportion of payment disparities, substantial disparities remain within several insurance subgroups. This finding suggests that eliminating payment disparities would also require narrowing gaps in payment generosity within insurance categories.

### Limitations

Our study has several limitations. First, we did not examine some components of costs and revenues that may be relevant for physician incentives. For costs, our primary estimates do not account for time or administrative burdens that differ despite similar visit intensity according to RVUs. However, payment disparities are unlikely to be alleviated by differences in clinician time costs, since visit length is very similar across patient race and ethnicity.^34^ Also, in sensitivity analyses, measured payment disparities were consistent despite alternate measures of visit intensity and cost and were larger when we accounted for administrative costs of revenue collection. If there were systematic differences across patient demographic groups in the number of RVUs billed for services with the same cost, this would also introduce bias in our estimates of payment disparities. For revenues, we excluded visits with capitation payments to physician practices; however, this sample restriction did not exclude visits with FFS payments from capitated insurers (eg, Medicare managed care plans). Because FFS payments remain the dominant form of payment to physician organizations,^35^ even at large health systems,^36^ studies quantifying payment generosity typically focus on FFS payments.^37^

Second, because of data limitations, our analysis excludes hospital-based care. Payment disparities may be larger for hospitals because of the larger differences between commercial insurers and Medicare for hospital payments than for physician payments^3^; supplemental hospital payments from Medicaid may counteract this phenomenon, however.^38^ Third, because we analyzed payments for completed visits, we could not observe any payment disparities large enough to prevent visits from occurring; this omission would likely contribute to underestimation of payment disparities. Fourth, because we studied payment disparities within geographic markets, we did not measure physician incentives to locate in markets with more racial and ethnic minority patients, another possible source of disparities in health care access. Finally, while our study quantifies the incentives for serving different patient groups, we do not directly measure the consequences of these incentives for patients or physician clinics. Still, prior research on the effects of increased payment generosity suggests that eliminating payment disparities could substantially shrink racial and ethnic disparities in health care utilization.^10,11^

## Conclusions

In this study, US physician practices were paid more for visits with non-Hispanic White patients than for visits with Black or Hispanic patients, which may contribute to disparities in health care access and quality. Knowledge of these payment disparities, which are larger in pediatrics and partially explained by insurance differences, could inform payment reforms addressing health care disparities.

## Notes

Supplement 1.
eAppendix.

eMethods.

eReferences.
**eTable 1.** Flow Chart of Study Sample **eTable 2.** Variable Sources/Definitions: Visit and Market Characteristics **eTable 3.** Variable Sources/Definitions: Insurance, Sociodemographic, and Health Characteristics **eTable 4.** Detailed Sample Characteristics by Racial/Ethnic Group **eTable 5.** Payment Disparities for Outpatient Visits by Subgroup **eTable 6.** Additional Decompositions of Payment Gaps **eTable 7.** Health Care Utilization Disparities With and Without Payment Disparities **eFigure.** Health Care Utilization Disparities With and Without Payment Disparities

Supplement 2. Data Sharing Statement
