# Supplementary material for: Payments to Physician Practices and Incentives to Serve Different Racial and Ethnic Groups
Source: JAMA Health Forum. 2025 Nov 26;6(11):e254561. doi: 10.1001/jamahealthforum.2025.4561 (PMC12658675; doi:10.1001/jamahealthforum.2025.4561)
Supplement: Supplement 1. — eAppendix. eMethods. eReferences. eTable 1. Flow Chart of Study Sample eTable 2. Variable Sources/Definitions: Visit and Market Characteristics eTable 3. Variable Sources/Definitions: Insurance, Sociodemographic, and Health Characteristics eTable 4. Detailed Sample Characteristics by Racial/Ethnic Group eTable 5. Payment Disparities for Outpatient Visits by Subgroup eTable 6. Additional Decompositions of Payment Gaps eTable 7. Health Care Utilization Disparities With and Without Payment Disparities eFigure. Health Care Utilization Disparities With and Without Payment Disparities [file jamahealthforum-e254561-s001.pdf]

# Supplemental Online Content

Schwartz AL, Asch DA, Werner RM. Payments to physician practices and incentives to serve different racial and ethnic groups. *JAMA Health Forum*. 2025;6(11):e254561. doi:10.1001/jamahealthforum.2025.4561

**eAppendix.**

**eMethods.**

**eReferences.**

**eTable 1.** Flow Chart of Study Sample

**eTable 2.** Variable Sources/Definitions: Visit and Market Characteristics

**eTable 3.** Variable Sources/Definitions: Insurance, Sociodemographic, and Health Characteristics

**eTable 4.** Detailed Sample Characteristics by Racial/Ethnic Group

**eTable 5.** Payment Disparities for Outpatient Visits by Subgroup

**eTable 6.** Additional Decompositions of Payment Gaps

**eTable 7.** Health Care Utilization Disparities With and Without Payment Disparities

**eFigure.** Health Care Utilization Disparities With and Without Payment Disparities

This supplementary material has been provided by the authors to give readers additional information about their work.

© 2025 Schwartz AL et al. *JAMA Health Forum*.

## eAppendix.

The following provides additional details regarding the conceptual motivation for our study, excluded for brevity from the main manuscript text.

**Possible sources of payment differences**

For a fee-for-service visit at a physician clinic, the total payment to the clinic depends on several factors. The content of a claim depends on visit content (i.e., the services provided), visit setting (i.e., hospital outpatient vs free-standing clinic), and coding intensity (i.e., the discretion of the billing provider in determining the service codes in the claim based on the visit content). The amount of payment for a given claim depends on fee generosity (i.e., the physician clinic's negotiated or administratively set rate with the payer), and any non-payment on the part of the insurance payer (i.e., denied claims) or patient (i.e., cost-sharing).

Any of these factors could contribute to differences in average payment amounts for encounters with different patient demographic groups. Several mechanisms are likely to be particularly important for fee generosity. Differences across demographic groups with respect to broad categories of health insurance coverage (i.e., Medicare Advantage vs Medicaid, etc.) will yield differences in fee generosity and claim denials.[1] Even within a single broad insurance segment (i.e. commercial insurance) within a single geographic area, fee generosity may be correlated with patient demographic characteristics. These differences could arise because of various features related to the price negotiation between insurers and provider organizations. Consider a commercial insurance plan with a narrow provider network and low premiums, which offers less generous fees to in-network physician clinics than competing insurers. This insurance plan would tend to attract beneficiaries with a lower willingness to pay for provider network breadth, which may be correlated with race or ethnicity.[2] It is also possible that price negotiations between physician clinics and insurers could be influenced by bias or discrimination against the demographic identity of a physician or against the identities of the patient population a clinic serves.

© 2025 Schwartz AL et al. *JAMA Health Forum*.

Our study examines some, but not all, of these possible sources of payment differences for clinic visits with different patient groups. Our primary analyses adjust payment differences for measures of visit content, geographic market, and year. We also exclude encounters at hospital outpatient departments, the primary source of increased payment due to visit setting. Thus, our analysis attempts to isolate payment differences associated with variation in fee generosity and non-payment within a given geographic market. Although we adjust payment differences for a variety of visit characteristics (e.g., RVUs billed, visit purpose, etc.), we cannot fully distinguish between coding intensity and visit content. Coding intensity and visit content are difficult to quantify independently because survey-obtained visit characteristics offer fairly limited additional data about visit content beyond the information coded in insurance claims (see eTable 2).

**Possible consequences of payment differences**

What might be the consequences of differences in payment levels for clinic encounters with patients from different racial/ethnic groups, adjusted for visit content, geography and year? These differences in provider payment levels are differences in supplier prices (as distinct from prices faced by patients, who are partially shielded from price via the mechanism of health insurance). Therefore, theory and evidence on how physician clinics respond to supplier prices can help forecast some potential consequences. Supplier prices affect the incentives facing physician clinics with respect to organizational decision-making. We use the term incentive broadly, referring to the financial force influencing all price-sensitive decisions made by physician clinics.

Differences in supplier prices might affect health care access, utilization, and quality in a variety of ways. Consider a primary care physician clinic that largely serves patients from racial/ethnic minority communities and receives relatively low fee-for-service payments on average because of the payer mix of these patients. Moreover, let us assume that this clinic is committed to treating all its patients similarly regardless of insurance status or demographics. This assumption illustrates the importance of financial

© 2025 Schwartz AL et al. *JAMA Health Forum*.

incentives even when clinics pursue non-monetary goals — a key concept in health economics since the field's inception.[3,4] If policy reforms were to level-up payment rates, eliminating payment disparities, then revenue would increase. The clinic may use that revenue to hire additional clinicians and support staff in ways that increase access, utilization and quality (e.g., offering more or longer appointment slots, responding more efficiently to patient telephone/electronic inquiries, etc.). If, on the other hand, policy reforms increased payment disparities substantially, revenue could decrease so much that administrators opt to close the clinic rather than deliver the lower quality health care afforded by the lower revenue. In this example, payment differences act as a resource constraint for the clinic, limiting access to health care and/or the resource-intensity of health care, exacerbating health care disparities.

A clinic may respond to payment differences in ways that differentially affect its patients from racial/ethnic minority groups. For example, in the presence of large payment differences, a clinic might refuse to accept insurance (i.e., accept in-network designation) below a certain threshold of payment generosity. Or, a clinic might choose to shrink services that are particularly valuable to patients with lower generosity insurance (e.g., translation services, social work). A clinic with multiple locations might shrink its capacity in a neighborhood that serves patients with lower insurance generosity; for patients in that neighborhood, reduced capacity might yield fewer available appointments, lapses in health care quality, and/or worse patient satisfaction. As these examples suggest, a policy reform that eliminates payment disparities by leveling-up payment rates may cause not only broad increases in health care production (i.e. from revenue gains allocated broadly across services) but targeted increases in the health care activities that now generate increased revenue.

Of course, there may be considerable variation across physician clinics in the extent to which they respond to financial incentives. However, an extensive research literature has examined the average responses of physician clinics to prices for physician services. Studies in this literature tend to show that, on average, higher prices lead to increased health care access,[5,6] utilization,[7–10] and quality.[7,10–13] Thus, if physician clinics receive lower payments for serving a particular patient population, it is reasonable to

© 2025 Schwartz AL et al. *JAMA Health Forum*.

expect these populations to face, on average, disadvantages with respect to health care access, utilization, and quality (i.e., health care disparities).

© 2025 Schwartz AL et al. *JAMA Health Forum*.

**eMethods.**

The following provides additional methodological details excluded for brevity from the main manuscript text. Details relate to the Medical Expenditure Panel Survey data, sample restrictions, variable sources and definitions, payment disparity measurement, sensitivity analyses, and a supplementary analysis of the role of payment disparities in racial/ethnic disparities in outpatient health care utilization.

**Data: The Medical Expenditure Panel Survey**

Our main data source was the 2014-2021 Medical Expenditure Panel Survey Household Component (MEPS-HC) and the MEPS Medical Provider Components (MEPS-MPC). The MEPS-HC is a widely used, nationally representative household survey of the US civilian non-institutionalized population conducted annually since 1996. The MEPS-HC collects detailed information on health care use, health status, health insurance, and other characteristics for every member of each household. The MEPS utilizes an overlapping panel design with a new panel starting each year and typically remaining for two calendar years. Prior to the COVID-19 pandemic, each respondent was interviewed in-person for five rounds covering two calendar years. During the COVID-19 pandemic two panels (panels 23 and 24) were extended for an additional two-calendar years for a total of 4 years.

The MEPS-MPC is the principal source of expenditure data on the MEPS public use files and served as the source of both our outcome and our main covariates detailing visit characteristics. Each year, hospitals and a sample of physician's practices seen by MEPS-HC sample members are contacted by telephone to add detailed charge and payment information for health care encounters reported in the MEPS-HC. Response rates for physician practices in the MEPS-MPC are typically above 80%. For each encounter, the billing offices of providers are asked to provide the line-item CPT-4/HCPCS procedure code, the line-item charge, the total charge for the visit, and all payments by source for the visit, including insurance payments and out-of-pocket amounts. The presence of these procedure codes allowed us to merge detailed information about each procedure from additional sources (i.e. 2014-2021 Centers for

© 2025 Schwartz AL et al. *JAMA Health Forum*.

Medicare and Medicaid Services Physician Fee Schedule Relative Value Files, 2014-2021 CMS Part B Summary Files, and 2014-2021 MarketScan databases). The residences of MEPS respondents are fully geocoded by ZIP-code. This information was used to merge in additional market-level information as described in eTable 2, eTable 3, and below.

**Sample Restrictions**

This section details our sample exclusion restrictions and their justification. Our initial sample began with all ambulatory visits reported in the 2014-2021 MEPS-HC. We then excluded all visits without matching MEPS-MPC data for that visit. Because MEPS-MPC payment data were not gathered for many MEPS-HC visits, this was the most consequential exclusion criterion, responsible for the majority of excluded ambulatory visits. We next excluded all visits in Veterans Health Administration facilities, Indian Health Service facilities, and military facilities. Ambulatory visits to hospital outpatient department and emergency departments were then excluded for two principal reasons. First, hospital facility-based care frequently involves both facility and professional fee payments complicating analyses of physician practice incentives. Second, a high percentage of the hospital-based encounters have one or more line-items with a missing CPT-4/HCPCS procedure code and these are not missing at random. After these exclusions, the remaining visits were physician office facilities.

We then excluded any encounter that was missing one or more line-item CPT-4/HCPCS procedure codes and any encounter for which every line-item procedure code did not match to either the CMS PFS Relative Value Files, the Medicare FFS Summary file, or the Marketscan outpatient file for that year as these are our principal sources of data on service intensity for the visit. We further excluded encounters financed by capitated arrangements, the small percentage of encounters financed by a global or bundled fee, and the small percentage of fee-for-service visits with incomplete payment information. We then restricted our sample to Evaluation and Management encounters (E&M) based on CPT-4/HCPCS codes 99201–99215, 99381–99429 and G0438-G0439. We also excluded encounters above the

© 2025 Schwartz AL et al. *JAMA Health Forum*.

99th percentile (weighted, 99.2% unweighted) of total payments because we lacked sufficient sample to account for these outliers in our model and because they are atypical E&M encounters. Finally, because we lacked sufficient sample to examine differences in payments levels for other racial/ethnic groups, we restricted our sample to non-Hispanic White, non-Hispanic Black, and Hispanic patients. eTable 1 details the effect of these sequential exclusion rules on sample size. Of note, sample size limitations precluded analysis of race and ethnicity separately. Among our sample of Hispanic patients, 2.4% (n=653) were also identified by survey as Black. In a sensitivity analysis, we redefined mutually exclusive race/ethnicity categories as White (non-Hispanic), Black, and Hispanic (non-Black) and obtained identical disparity estimates (Table 3)

**Variable Definitions**

Minimal data processing steps were required to generate most of the variables employed in our analyses (see eTables 1 and 2). Here we provide additional details regarding the methods we used to generate variables that were more complex.

Because of rare data missingness, our primary analysis included three variables to quantify the intensity procedures codes billed for each visit. Our primary metric of service intensity was the aggregate RVU weight for a visits' billed services. However, these RVUs were not present in the corresponding annual CMS RVU for 3.1% of total line-item charges, with some non-matching line-item procedures in 10.2% of all visits. To account for the service intensity of the remaining procedure codes that could not be matched to the CMS RVU dataset, we generated a secondary variable to measure service intensity, equal to the total of the non-matching line-item charges obtained from the MEPS billing survey; this variable was set to zero for every visit for which all billed procedure codes were matchable to CMS RVUs. In a sensitivity analysis, we quantified the intensity of each visit's billed services by summing each line-item procedure code's mean payment in the MarketScan dataset corresponding to the visit's year.

© 2025 Schwartz AL et al. *JAMA Health Forum*.

For market characteristics, we supplemented regional geographic indicators with ZIP-code level measures of primary care physician practice concentration and a ZIP-code level measure of multispecialty practice concentration, as implemented in Meille et al. (2024). The construction of each market concentration variable proceeds in three steps from the Medicare 5% Fee for Service Carrier files for each study year. First, we calculated the traditional Herfindahl-Hirschman Index (HHI) for each patient ZIP by estimating market shares using the allowed charges on the Medicare Carrier FFS file and summing the squared market shares multiplied by 10,000. Next, we created firm-level HHIs by taking the average of the patient ZIP-code level HHIs, weighted by the share of the firm's claim allowed amounts from each ZIP-code. Finally, we created the final Kessler-McClellan adjusted HHI by taking the average of the firm HHIs in the prior step, weighted by each firm's market share in the ZIP-code.

Our main measure of health insurance coverage was a seven-category hierarchal, mutually exclusive set of indicators for the reported health insurance coverage at the time of the E&M encounter (See eTable 3). However, in analyses of visit subgroups defined by insurance source, we combined insurance categories into four broader, mutually exclusive and collectively exhaustive categories of insurance: any Medicare insurance, private insurance without Medicare, Medicaid only, or uninsured.

**Measuring Payment Disparities**

To measure payment disparities, we employed similar Kitagawa-Oaxaca-Blinder (KOB) methods previously used to measure health care disparities according to the Institute of Medicine's disparities definition.[14] These KOB methods entail four key methodological choices: defining the primary outcome, defining allowable sources of outcome variation, defining unallowable (i.e. disparity-contributing) sources of outcome variation, and selecting a specification for modelling outcomes as a function of these variables. Our main dependent variable was the total payment for a visit received by office-based practice from all sources including out-of-pocket payments, Medicare, Medicaid, private insurance, Tri-Care, and

© 2025 Schwartz AL et al. *JAMA Health Forum*.

all other sources of payments. We adjusted all years to 2021 dollars using National Health Expenditure Accounts Physician Price index.

Payment disparities can be defined as differences across patient racial/ethnic groups in physician practice payments, which do not arise from differences in the cost of delivering health care services. Importantly, when defined this way, payment disparities also can quantify the differential incentive for physician organizations to serve patients from one racial/ethnic group relative to another. (Of note, incentives depend on the amount of payment for serving additional patients from a racial/ethnic group; the payment amount for serving an additional, marginal patient may differ from the average payment amount for patients already being served by a physician practice).

Because the cost of providing a health care encounter is not directly observable, operationalizing a definition of payment disparities that accounts for cost differences requires proxy measures for cost. We followed the approach of the CMS resource-based relative value scale (RBRVS) for physician payment, which uses granular data on visit content for this purpose (i.e. detailed procedure codes for services delivered, and indicators for physician versus non-physician provider type), and also adjusts payments according to geographic region and year.[15] The economic rationale for adjusting a resource-based payment system for visit content, location, and year is straightforward; encounter costs depend on the quantities of required production inputs (e.g. clinician time), which vary according to the specific services provided, and on input prices (e.g. wages, rents), which vary by geographic market and over time.

Our primary model accounted for six broad sources of variation in total payment per visit. In our primary analysis, the three allowable sources of payment differences were year, visit characteristics (e.g provider type, visit type and service intensity), and geographic market characteristics; again, these constitute the main sources of payment adjustments in administrative fee-for-service payment systems because these factors all contribute to the input costs of professional services for a visit at a physician practice. The three other sources of payment variation which we modeled were patients' insurance

© 2025 Schwartz AL et al. *JAMA Health Forum*.

characteristics, sociodemographic characteristics, and health characteristics. See eTables 2-3 for additional details on variable source/definition. Inclusion of these (non-allowable) sources of payment variation in our regression model is necessary to address confounding in disparity estimates.[1]

Of note, previous methods for quantifying disparities in health care utilization (e.g. count of physician visits per year) categorize health status as an allowable source of variation in health care utilization.[14] However, in our primary analysis, we do not include health status within the set of allowable sources of variation in payment amounts. Our decision follows standard physician payment policies, which adjust fee-for-service payment rates according to visit content and geographic market characteristics, rather than according to the health status of the patient.

Still, because various definitions of payment disparities may be appropriate, we also measured unadjusted payment gaps and payment gaps adjusted for different sets of characteristics (e.g., health status) in secondary analyses. For example, consider that our primary analysis adjusts for allowable differences in payment levels across geographic areas. This quantity reflects financial incentives facing a given physician practice to serve patients from different racial/ethnic groups within the practices' geographic catchment area, which is the primary focus of our study. But, this quantity does not reflect incentives to locate in markets with more racial/ethnic minority patients, another possible source of health care access disparities. So, in additional decompositions, we varied what factors were categorized as allowable sources of payment differences (Table 2 and eTable 6).

Our estimates of payment disparities were derived from a pooled KOB linear regression model. Pooled regressions included visit data from all racial/ethnic patient groups. The greater sample size afforded by pooled modeling allowed greater precision in estimating coefficients for variables associated with relatively few visits (most importantly, the set of 266 indicators for an individual hospital referral region). Linear modeling avoided the computational intensity involved in nonlinear decomposition or

© 2025 Schwartz AL et al. *JAMA Health Forum*.

rank-and-replace ordinal postestimation calculations.[1] We used ordinary least squares regression to estimate the following model predicting total payment levels:

$$Y_i = \tau_i + \beta_0 VIS_i + \beta_1 MKT_i + \beta_2 INS_i + \beta_3 SDEMO_i + \beta_4 HLTH_i + \epsilon_i$$

**Equation 1**

where *i* denotes visit, $\tau$ are year fixed effects, *VIS* represents a vector of visit characteristics, *MKT* represents a vector of market characteristics, *INS* represents a vector of insurance characteristics, *SDEMO* represents a vector of sociodemographic characteristics and *HLTH* represents a vector of health characteristics. Of note, *SDEMO* contains an indicator for patient racial/ethnic group (non-Hispanic White, non-Hispanic Black, or Hispanic); the estimated coefficients for these indicators reflect payment differences across patient race/ethnicity that are not explained by other factors included in the model. All variable definitions and sources are described in eTables 2 and 3, with mean values presented in eTable 4.

To estimate payment disparities for visits with racial/ethnic group *g* relative to visits with non-Hispanic White patients, we calculated the ratio $\widehat{GAP}_g = \frac{\hat{Y}_g - \hat{Y}_{white}}{\hat{Y}_{white}}$ which represents the relative payment rate reduction for racial/ethnic group *g*, adjusted for allowable sources of payment differences. $\hat{Y}_g$ is the mean predicted payment across the $n_g$ count of visits with racial/ethnic group *g*, standardized to account for allowable sources of payment differences between racial/ethnic groups:

$$\hat{Y}_g = \frac{\sum_{i \in g} \bar{\tau}_{white} + \widehat{\beta_0}\, \overline{VIS}_{white} + \widehat{\beta_1}\, \overline{MKT}_{white} + \widehat{\beta_2} INS_i + \widehat{\beta_3} DEMO_i + \widehat{\beta_4} HLTH_i + \widehat{\beta_5} SES_i}{n_g}$$

**Equation 2**

This calculation adjusts expected payment amounts for visits with non-Hispanic Black and Hispanic patients by setting allowable sources of payment variation (i.e. visit characteristics, market characteristics, and year) equal to the mean value of these variables for visits with non-Hispanic white patients. Standard errors for estimated payment disparities were calculated using the delta method. In addition to our

© 2025 Schwartz AL et al. *JAMA Health Forum*.

primary estimate of payment disparities, we estimated payment disparities within visit subgroups using coefficients derived from our main pooled model (Figure 2 and eTable 5).

In decomposition analyses, we modified our calculations of $\widehat{GAP_g}$ by changing which variables were classified as allowable sources of payment differences across racial/ethnic groups (Table 2 and eTable 6). We re-estimated payment gaps adjusted for broader or narrower sets of allowable sources of payment differences across patient race/ethnicity (e.g. year, visit characteristics, market characteristics, and insurance characteristics). These modifications entail changing which variables in equation 2 are set to equal the mean values for the non-Hispanic White group. Note that all decomposition estimates are derived from results of the same regression model.

All results were adjusted for survey weights. We used a two-stage non-response adjustment at the event level to account for exclusions made for missing data. The first stage accounted for observations lost because no matching MEPS-MPC data were available for that visit. Matching MPC data may be unavailable because the patient did not sign an authorization form allowing the MEPS-MPC to contact their physician's office, the physician practice did not respond to the MEPS-MPC, or no matching MEPS-MPC data could be found for that MEPS-HC reported encounter. To calculate survey weights, we began first with the MEPS public use file sample weights and then estimated the probability of each event matching to the MEPS-MPC via probit regression modelling. Controls include the standard variables used in the development of the MEPS weights (age, sex, race/ethnicity, income, education, Census region, MSA status), variables used to sample office-based visits for the MEPS-MPC, and visit characteristics. We used a hybrid approach to develop this 1st stage non-response adjustment,[16] sorting all observations by their predicted probability of having MEPS-MPC data, dividing the observations into 20 equal cells, and then rescaling the weights of the observations with MEPS-MPC data within each cell so that the sum of weights in that cell equals the sum of weights of all observations (missing and non-missing) in that cell.

© 2025 Schwartz AL et al. *JAMA Health Forum*.

In the second stage, we used this same hybrid non-response approach for the probability of having complete payment and procedure codes conditional on matching to the MEPS-MPC. Controls for this second stage include all controls included in the first stage as well as deciles of total charge. Separate non-response adjustments are made in both stages for each year. Although no raking was applied in either stage due to lack of adequate external control totals, the marginal distributions of our final sample using our two-stage non-response adjusted weight matches the distributions of the initial sample using the original MEPS public use file weights based on age, sex, race/ethnicity, income, education, Census region, and MSA status.

We used this adjusted survey weight in all analyses. All analyses were conducted using STATA/MP 18 and corrected for the complex multistage clustered and stratified design of the MEPS using the STATA svy commands, which also account for the repeated observations in the sample.[17,18] All differences discussed in the text were statistically significant at the p=.05 level or lower, unless otherwise noted.

**Sensitivity Analyses**

To assess the robustness of our payment disparity estimates, we conducted several sensitivity analyses. For each sensitivity analysis, we modified one feature of our primary analysis. First, we deflated our primary outcome, total payment from all sources, to account for the administrative and time costs that physician offices face from claims denials and resubmissions, which differ by insurance payer.[1] Based on previously published estimates of physician claim resubmission costs,[19] we deflated total visit payments by 3.84% for patients with Medicaid, 1.45% for patients with Medicare and 0.51% for patients with commercial insurance. Next, we replaced RVU-based measures of service intensity with measures based on each procedure's standardized price in Marketscan, as described above. Next, we measured service intensity using the RVU physician work component rather than total RVUs. Next, we modeled RVUs using a piecewise spline rather than a polynomial. Next, we used more detailed physician specialty

© 2025 Schwartz AL et al. *JAMA Health Forum*.

categories than the three-tiered measure employed in our primary model; in the sensitivity analysis, we included binary indicators for the following specialties: allergy/immunology, anesthesiology, cardiology, dermatology, endocrinology, family practice, gastroenterology, general practice, general surgery, geriatrics, obstetrics/gynecology, hematology, internal medicine, nephrology, neurology, oncology, ophthalmology, orthopedics, osteopathy, otorhinolaryngology, pediatrics, physical medicine and rehabilitation, plastic surgery, psychiatry, pulmonology, radiology, rheumatology, urology, other, or specialty missing.

Next, instead of HRRs, we defined patient geographic region indicators using 106 CMS Geographic Price Index regions, which are used to adjust Part B physician fees regionally. Next, we added several variables potentially correlated with private insurance generosity including establishment size, multilocation firm, whether private coverage was obtained through a current employer, and whether the person was the policyholder. Next, we included a binary indicator for residence in a state where Medicaid pays the Medicare cost-sharing amount in full, regardless of what their Medicaid rate is for the service vs. a "lesser-than" policy state. Next, we included the outlier encounters above the 99th percentile of total visit charges. Next we restricted the sample to encounters for practices participating in the 2015-2016 MEPS Medical Organizations Survey (n=29,943 visits) and added the following physician practice characteristics: number of doctors (1, 2-3, 4-10, >10), multispecialty practice, multiple locations, practice type (independent, government or non-profit, system). Finally, we modeled visit payments with a three-way model allowing different estimated coefficients on included variables for each racial/ethnic groups. As is standard, a minoritized group's coefficients was taken as the reference for calculating adjusted payment amounts for that minoritized group.

**Supplementary Analysis of Payment Disparities and Disparities in Health Care Use**

Although we cannot directly measure the effects of payment disparities on racial/ethnic disparities in health care, we can indirectly approximate the magnitude of these effects based on findings

© 2025 Schwartz AL et al. *JAMA Health Forum*.

from prior studies. To conduct this supplementary analysis, we drew from previously published estimates of the price elasticity of clinician supply, a measure of how clinician payment levels affect health care utilization.[8,9] Specifically, the price elasticity of clinician supply measures the percent increase in health care utilization that occurs for any percent increase in clinician fees. We used elasticity estimates to project how racial/ethnic health care utilization disparities would change if the racial/ethnic payment disparities we measured were eliminated. The main assumption of this analysis is that price changes from eliminating payment disparities would produce the same increases in health care utilization as measured in the prior studies.

First, we quantified existing disparities in outpatient health care utilization by race/ethnicity using MEPS utilization data. For MEPS respondents who were White (non-Hispanic), Black (non-Hispanic) or Hispanic, we calculated the annual counts of visits using the MEPS office-based medical provider visit file and outpatient department visit file. For all utilization outcomes, we measured only outpatient visits with physicians, and excluded emergency department visits. We measured not only the total count of health care encounters per year, but, based on survey-obtained reasons for the visit, we also measured the total count of general checkups per year. Because we had observed larger payment disparities for visits with pediatric patients, we separately estimated annual visit rates for children aged 17 or under, and also measured annual rates of pediatric mental health encounters.

Next, we quantified existing racial/ethnic disparities in annual physician visit rates using KOB methods for measuring health care disparities according to the Institute of Medicine definition of health care disparities. We modeled visit rates using pooled ordinary least squares regression, with covariates for allowable (age, gender and health status) and non-allowable sources of utilization differences (race/ethnicity, health insurance coverage, household size, marital status, education, income, language, county level poverty and county level racial and ethnic composition). We then calculated adjusted visit rates for Black (non-Hispanic) patients and Hispanic patients, accounting for group differences in age,

© 2025 Schwartz AL et al. *JAMA Health Forum*.

gender and health status by predicting visit rates based on the average age, gender and health status characteristics observed among the non-Hispanic White patients.

Finally, using previously published estimates of physician supply elasticity, we estimated how adjusted utilization rates would increase if payment disparities were eliminated. Separately for Black (non-Hispanic) and Hispanic patients, we estimated the adjusted visit rate without payment gaps as:

$$Q_g^{new} = Q_g^{gap} + P_g * \varepsilon * Q_g^{gap}$$

where $Q_g^{gap}$ is the adjusted visit rate for patients in racial/ethnic group $g$ in the presence of payment disparities, $P_g$ is the payment advantage for visits with White patients relative to visits with patients in racial/ethnic group $g$, calculated as $\frac{\hat{Y}_{white} - \hat{Y}_g}{\hat{Y}_g}$ and $\varepsilon$ is the price elasticity of physician supply. For outcomes related to children, we used payment disparity estimates from our subgroup analysis of visits with patients aged 17 or younger. To demonstrate how our results are sensitive to the choice of elasticity estimates, we used two different published elasticity estimates in our calculations. The first elasticity, 1.18, was estimated based on changes in evaluation and management visits during the two years after payment increases for Medicaid primary care services.[8] The second elasticity, 1.46, was estimated based on changes in aggregate health care utilization 5-9 years after exogenous changes to Medicare physician payment levels.[7] These elasticity estimates indicate that a 10% increase in physician payments leads to a more than 10% increase in health care utilization. For example, if Hispanic children had 1 checkup per year in the presence of payment disparity $P_g$ of 10%, then with an elasticity of 1.18, we would expect that eliminating the payment disparity would yield a $Q_g^{new}$ of 1 + 0.1 * 1.18 * 1= 1.118 checkups per year.

The results of this analysis are presented in eTable 7 and eFigure 1. The largest disparities in health care utilization occurring during the study period were in pediatric mental health visits. Adjusted comparisons showed that these visits occurred 64% less than the non-Hispanic White mean for non-Hispanic Black children, and 53% less than the non-Hispanic White mean for Hispanic children.

© 2025 Schwartz AL et al. *JAMA Health Forum*.

Disparities were smallest for pediatric general checkups, though they were still substantial. Pediatric general checkups occurred 31% less than the non-Hispanic White mean for non-Hispanic Black children, and 24% less than the non-Hispanic Black mean for Hispanic children.

Utilization disparities were projected to narrow substantially in the absence of payment disparities, though the extent of narrowing varied across utilization measures and across elasticity estimates. With an elasticity of 1.18, the elimination of payment disparities was projected to narrow Black–White utilization disparities by 7–13 percentage points and Hispanic–White utilization disparities by 8–16 percentage points, depending on the utilization measure. With an elasticity of 1.46, the elimination of payment gaps was projected to narrow Black–White utilization disparities by 8–16 percentage points and Hispanic–White utilization disparities by 9–20 percentage points, depending on the utilization measure. For example, with the larger elasticity, elimination of payment disparities was projected to reduce the size of the Hispanic–White disparity in pediatric general checkups from 24% to 4.7% of the non-Hispanic White mean.

© 2025 Schwartz AL et al. *JAMA Health Forum*.

## eReferences.


1. Gottlieb JD, Shapiro AH, Dunn A. The Complexity Of Billing And Paying For Physician Care. *Health Aff (Millwood)*. 2018;37(4):619-626. doi:10.1377/hlthaff.2017.1325

2. Rae M, Pollitz K, Pestaina K, Long M, Lo J, Published CC. How Narrow or Broad Are ACA Marketplace Physician Networks? KFF. August 26, 2024. Accessed July 30, 2025. https://www.kff.org/private-insurance/report/how-narrow-or-broad-are-aca-marketplace-physician-networks/

3. Newhouse JP. Toward a Theory of Non-Profit Institutions: An Economic Model of a Hospital. *Am Econ Rev*. 1970;60:64-74.

4. Arrow KJ. Uncertainty and the Welfare Economics of Medical Care. *Am Econ Rev*. 1963;53(5):941-973.

5. Saulsberry L, Seo V, Fung V. The Impact of Changes in Medicaid Provider Fees on Provider Participation and Enrollees' Care: a Systematic Literature Review. *J Gen Intern Med*. 2019;34(10):2200-2209. doi:10.1007/s11606-019-05160-x

6. Polsky D, Richards M, Basseyn S, et al. Appointment Availability after Increases in Medicaid Payments for Primary Care. *N Engl J Med*. 2015;372(6):537-545. doi:10.1056/NEJMsa1413299

7. Clemens J, Gottlieb JD. Do Physicians' Financial Incentives Affect Medical Treatment and Patient Health? *Am Econ Rev*. 2014;104(4):1320. doi:10.1257/aer.104.4.1320

8. Cabral M, Carey C, Miller S. The Impact of Provider Payments on Health Care Utilization of Low-Income Individuals: Evidence from Medicare and Medicaid. *Am Econ J Econ Policy*. Published online Forthcoming.

9. Brekke KR, Holmås TH, Monstad K, Straume OR. Do treatment decisions depend on physicians' financial incentives? *J Public Econ*. 2017;155:74-92. doi:10.1016/j.jpubeco.2017.09.012

10. Dunn A, Shapiro AH. Physician Competition and the Provision of Care: Evidence from Heart Attacks. *Am J Health Econ*. 2018;4(2):226-261. doi:10.1162/ajhe_a_00099

11. Shen YC, Zuckerman S. The Effect of Medicaid Payment Generosity on Access and Use among Beneficiaries. *Health Serv Res*. 2005;40(3):723-744. doi:10.1111/j.1475-6773.2005.00382.x

12. Brunt CS, Jensen GA. Medicare Part B reimbursement and the perceived quality of physician care. *Int J Health Care Finance Econ*. 2010;10(2):149-170. doi:10.1007/s10754-009-9075-1

13. Alexander D, Schnell M. The Impacts of Physician Payments on Patient Access, Use, and Health. *Am Econ J Appl Econ*. 2024;16(3):142-177. doi:10.1257/app.20210227

14. Cook BL, McGuire TG, Zaslavsky AM. Measuring Racial/Ethnic Disparities in Health Care: Methods and Practical Issues. *Health Serv Res*. 2012;47(3pt2):1232-1254. doi:10.1111/j.1475-6773.2012.01387.x

15. Physician and Other Health Professional Payment System. Published online November 2021. https://www.medpac.gov/wp-content/uploads/2021/11/medpac_payment_basics_21_physician_final_sec.pdf


© 2025 Schwartz AL et al. *JAMA Health Forum*.

16. Valliant R, Dever JA, Kreuter F. *Practical Tools for Designing and Weighting Survey Samples*. Springer International Publishing; 2018. doi:10.1007/978-3-319-93632-1

17. Williams RL. A Note on Robust Variance Estimation for Cluster-Correlated Data. *Biometrics*. 2000;56(2):645-646.

18. Accounting for Clustering in the Analysis of MEPS Data: Frequently Asked Questions (FAQ). Accessed October 17, 2024. https://meps.ahrq.gov/survey_comp/hc_clustering_faq.pdf

19. Dunn A, Gottlieb JD, Shapiro AH, Sonnenstuhl DJ, Tebaldi P. A Denial a Day Keeps the Doctor Away*. *Q J Econ*. 2024;139(1):187-233. doi:10.1093/qje/qjad035

© 2025 Schwartz AL et al. *JAMA Health Forum*.

**eTable 1.** Flow Chart of Study Sample

| | Sample size remaining | | | | | | Sample loss from previous step | | | |
|---|---|---|---|---|---|---|---|---|---|---|
| | White | Black | Hispanic | Asian | Other | Total | White | Black | Hispanic | Total |
| Initial Sample -- All MEPS - MPC Eligible Hospital Outpatient Department and Physician Office Visits as Reported by the Household (2014-2021) | 683,041 | 169,402 | 207,439 | 43,871 | 41,499 | 1,145,252 | | | | |
| Include only those that match to MEPS-MPC[a] | 303,027 | 67,402 | 78,899 | 15,185 | 17,743 | 482,256 | 55.6% | 60.2% | 62.0% | 57.9% |
| Drop VA, IHS and Military Health Facilities | 291,427 | 63,101 | 77,293 | 14,958 | 16,159 | 462,938 | 3.8% | 6.4% | 2.0% | 4.0% |
| Restrict to office-based doctors based on MPC data collection[b] | 172,413 | 36,706 | 50,078 | 9,365 | 9,205 | 277,767 | 40.8% | 41.8% | 35.2% | 40.0% |
| Drop if missing 1 or more CPT4 codes | 155,828 | 33,324 | 44,634 | 8,528 | 7,906 | 250,220 | 9.6% | 9.2% | 10.9% | 9.9% |
| Drop if missing any payment data[c] | 151,443 | 31,325 | 40,985 | 8,160 | 7,578 | 239,491 | 2.8% | 6.0% | 8.2% | 4.3% |
| Drop if capitated payment | 146,669 | 29,559 | 35,653 | 7,271 | 7,323 | 226,475 | 3.2% | 5.6% | 13.0% | 5.4% |
| Drop if global fee/bundled payment | 142,985 | 29,058 | 34,934 | 7,121 | 7,191 | 221,289 | 2.5% | 1.7% | 2.0% | 2.3% |
| Restrict to E & M Visits | 104,124 | 22,218 | 27,253 | 5,571 | 5,467 | 164,633 | 27.2% | 23.5% | 22.0% | 25.6% |
| Drop observations above 99% percentile of total charges (weighted) | 103,185 | 22,036 | 27,115 | 5,542 | 5,408 | 163,286 | 0.9% | 0.8% | 0.5% | 0.8% |
| Drop Asian and Other Race/Ethnicity categories | 103,185 | 22,036 | 27,115 | 0 | 0 | 152,336 | 0.0% | 0.0% | 0.0% | 6.7% |

[a] Non-response adjustment applied to MEPS sample weights to account for missing MPC data. Reasons MPC data may be missing include (1) MEPS sample member did not sign authorization form, (2) Office-based providers are subsampled in the MEPS-MPC data collection, (3) The provider could not be located, (4) The provider did not respond to the MEPS-MPC, (5) The data collected from the provider in the MEPS-MPC could not be matched back to data reported by the MEPS-HC respondent.

[b] a large fraction of providers identified by household respondents as office based/clinic based end up being identified as hospital-based during MPC data collection. A smaller fraction identified by households as hospital-based events turn out to be physician office-based events

[c] Second adjustment applied to weights to account for missing CPT4 and/or payment data

© 2025 Schwartz AL et al. *JAMA Health Forum*.

**eTable 2.** Variable Sources/Definitions: Visit and Market Characteristics

**Visit Characteristics**

| Characteristic | Source | Notes |
|---|---|---|
| Visit RVUs | MEPS MPC & Medicare RVU Files | Total RVUs are based on billed CPT-4/HCPCS codes and include work, practice and malpractice components. Our primary model includes both RVUs and RVUs-squared. In a sensitivity analysis, work component RVUs are used rather than total RVUs. |
| Medicare Part B average allowed charges | MEPS MPC & Medicare Part B National Summary File | Allowed charges include insurer-paid and out-of-pocket charges |
| Total charges | MEPS MPC | Defined as the continuous, visit-level total charges as reported in the MPC survey. Defined as zero for all visits for which every CPT-4/HCPCS code was matchable to Medicare RVU files, the Medicare Part B National Summary File, or MarketScan Outpatient File. |
| Purpose of visit | MEPS Household Component (HC) | Characterized with the following mutually exclusive indicators: (1) general checkup, (2) diagnosis or treatment, (3) emergency, (4) psychotherapy or mental health counseling, (5) follow-up or post-operative, (6) immunizations/shots (7) vision exam, (8) pregnancy-related, (9) well child exam, (10) other, (11) missing |
| Clinician type | MEPS HC | Categorized as primary care physician, specialty physician, or non-physician provider |
| Physician specialty | MEPS HC | Physician specialties categorized in three tiers assigned based on expected payment levels as developed by AHRQ for editing and imputing MEPS expenditure data. |
| Laboratory testing | MEPS HC | Indicator for any laboratory testing associated with the visit |
| Radiologic testing | MEPS HC | Indicator for any radiographic testing associated with the visit |
| Federally qualified health center or rural health center | MEPS HC | Indicator for the visit occurring at a Federally Qualified Health Center or rural health clinic |

**Market Characteristics**

| Characteristic | Source | Notes |
|---|---|---|
| Hospital referral region | Dartmouth Atlas | Indicators for each of 266 regions. HRRs with less than 25 observations were collapsed into adjacent HRRs. |
| Zip-code physician concentration | Meille et al., 2024 | Kessler-McClellan adjusted zip-code level primary care physician practice Herfindahl-Hirschman Index (HHI) as implemented in Meille et al. 2024 using CMS administrative data. |
| Zip-code multispecialty firm concentration | Meille et al., 2024 | Kessler-McClellan adjusted zip-code level multispecialty practice Herfindahl-Hirschman Index (HHI) as implemented in Meille et al. 2024 using CMS administrative data. |
| Geographic Practice Cost Index region | CMS | Regional indicators for 106 regions, which are used for CMS geographic payment adjustments, was used instead of hospital referral region in a sensitivity analysis |
| Geographic Practice Cost Index work component | CMS | |
| Geographic Practice Cost Index practice expense component | CMS | |
| Geographic Practice Cost Index malpractice component | CMS | |

© 2025 Schwartz AL et al. *JAMA Health Forum*.

**eTable 3.** Variable Sources/Definitions: Insurance, Sociodemographic, and Health Characteristics

**Insurance Characteristics**

| Characteristic | Source | Notes |
|---|---|---|
| Insurance source | MEPS Household Component (HC) | Characterized using the following mutually exclusive indicators : (1) Medicare Only, (2) Medicare and private insurance coverage, (3) Medicare and Medicaid (dual eligibles), (4) Private insurance only (omitted), (5) Private and Medicaid coverage, (6) Medicaid only, and (7) uninsured |
| Medicare Advantage | MEPS HC | Binary indicator |
| Medicaid HMO | MEPS HC | Binary indicator |
| Private HMO | MEPS HC | Binary indicator |
| Lesser-of state vs. Full-payment | MACPAC | Binary indicator for whether state Medicaid pays the Medicare cost-sharing amount in full, regardless of what their Medicaid rate is for the service. This variable is used in sensitivity analyses. |
| Employment characteristics | MEPS HC | Establishment size (1-49, 50-99, 100-499,500+); firm has multiple locations; person is policyholder; insurance is through current main job). Used in sensitivity analyses. |

**Sociodemographic Characteristics**

| Characteristic | Source | Notes |
|---|---|---|
| Age | MEPS HC | Categorically defined as 0-4, 5-17, 18-24, 25-44, 45-64, 65-74, 75-84 and 85 or older |
| Gender | MEPS HC | Categorized as male or female based on household report |
| Family size | MEPS HC | Count of number of members in family |
| Marriage status | MEPS HC | Binary Indicator |
| Highest educational attainment in household | MEPS HC | Maximum categorized as high school diploma, some college, bachelor's degree, masters degree or greater |
| Family income | MEPS HC | Categorized relative to FPL level of the interview year: <100%FLP, 100-125% FPL, 125-200% FPL, 200-400% FPL, 400%+ FPL |
| Language | MEPS HC | Indicator for non-English survey interview |
| County % non-Hispanic White | AHRQ Social Determinants of Health Database | County proportion of non-Hispanic White residents. |
| County % below poverty | AHRQ Social Determinants of Health Database | County proportion of households with incomes below 100% FPL. |

© 2025 Schwartz AL et al. *JAMA Health Forum*.

**Health Characteristics**

| Characteristic | Source | Notes |
|---|---|---|
| AHRQ Priority conditions count | MEPS HC | Two variables employed; condition count and binary indicator for three or more conditions. Priority conditions were ascertained by asking respondent whether doctor ever told you/family member that you had: coronary heart disease, angina, myocardial infarction, and other unspecified heart disease, stroke, emphysema, high cholesterol, cancer, arthritis, diabetes, asthma, joint pain, or chronic bronchitis. |
| Any activity limitation | MEPS HC | Binary indicator for whether the household respondent reported any limitation in work, housework, or school. |
| Any Activity of Daily Life (ADL) limitation | MEPS HC | Binary indicator for whether the MEPS respondent reported the person "receive help or supervision with personal care such as bathing, dressing, or getting around the house (because of an impairment or a physical or mental health problem)?" |
| Perceived health status | MEPS HC | Categorized as excellent, very good, good, fair or poor |
| Priority for oversampling office-based providers in the MEPS-MPC | MEPS HC | Binary indicator for whether MEPS sample member (1) had an inpatient stay, (2) any home health care, (3) died, or (4) was institutionalized part of the year. |

© 2025 Schwartz AL et al. *JAMA Health Forum*.

**eTable 4.** Detailed Sample Characteristics by Racial/Ethnic Group

| Patient sociodemographic characteristics | Non-Hispanic White | | Non-Hispanic Black | | Hispanic | |
|---|---|---|---|---|---|---|
| | Mean | Standard Error | Mean | Standard Error | Mean | Standard Error |
| Age | | | | | | |
| 0—4 (%) | 5.4% | 0.3% | 5.7% | 0.5% | 11.7% | 0.7% |
| 5—17 (%) | 8.4% | 0.3% | 9.7% | 0.6% | 18.1% | 0.8% |
| 18—24 (%) | 4.6% | 0.2% | 5.7% | 0.7% | 6.7% | 0.5% |
| 25—44 (%) | 17.3% | 0.5% | 19.8% | 1.0% | 19.6% | 0.9% |
| 45—64 (%) | 29.5% | 0.5% | 35.3% | 1.1% | 26.8% | 1.1% |
| 65—74 (%) | 18.9% | 0.5% | 13.8% | 0.8% | 10.7% | 0.8% |
| 75—84 (%) | 12.0% | 0.4% | 7.6% | 0.6% | 4.9% | 0.4% |
| 85+ (%) | 3.9% | 0.2% | 2.5% | 0.5% | 1.5% | 0.2% |
| Male (%) | 41.9% | 0.5% | 35.6% | 1.2% | 42.3% | 1.0% |
| Female (%) | 58.1% | 0.5% | 64.4% | 1.2% | 57.7% | 1.0% |
| Family size | 2.493 | 0.022 | 2.404 | 0.041 | 3.258 | 0.049 |
| Married (%) | 49.7% | 0.6% | 27.8% | 1.2% | 35.2% | 1.1% |
| Household highest education attainment | | | | | | |
| Less than high school diploma (%) | 2.7% | 0.2% | 8.0% | 0.8% | 13.5% | 0.9% |
| High school diploma (%) | 18.1% | 0.6% | 27.4% | 1.2% | 23.5% | 1.1% |
| Some college (%) | 26.4% | 0.7% | 30.3% | 1.3% | 28.4% | 1.1% |
| Bachelors or equivalent (%) | 26.4% | 0.6% | 19.0% | 1.3% | 22.2% | 1.3% |
| Masters or greater (%) | 26.3% | 0.8% | 15.3% | 1.2% | 12.4% | 1.0% |
| Family income | | | | | | |
| Family income < FPL (%) | 8.7% | 0.4% | 22.9% | 1.0% | 20.5% | 1.3% |
| Family income 100-125% of FPL (%) | 3.3% | 0.2% | 6.9% | 0.6% | 6.8% | 0.5% |
| Family income 125-200% of FPL (%) | 10.8% | 0.4% | 16.2% | 1.0% | 18.0% | 0.9% |
| Family income 200-400% of FPL (%) | 26.3% | 0.5% | 27.3% | 1.1% | 28.4% | 1.2% |
| Family income > 400% FPL (%) | 50.9% | 0.8% | 26.8% | 1.4% | 26.2% | 1.4% |
| Non-English language interview | 0.4% | 0.1% | 0.4% | 0.1% | 37.7% | 1.6% |
| County % non-Hispanic White | 74.84 | 0.46 | 43.76 | 0.99 | 43.09 | 1.78 |
| County % < FPL | 12.26 | 0.20 | 19.08 | 0.35 | 17.36 | 0.78 |

| Patient health characteristics | Non-Hispanic White | | Non-Hispanic Black | | Hispanic | |
|---|---|---|---|---|---|---|
| | Mean | Standard Error | Mean | Standard Error | Mean | Standard Error |
| Activity limitations | | | | | | |
| Any activity limitation (%) | 18.0% | 0.5% | 24.9% | 1.1% | 16.4% | 1.1% |
| Any ADL limitation (%) | 14.7% | 0.5% | 21.5% | 1.2% | 13.2% | 0.9% |
| Health status | | | | | | |
| Excellent (%) | 21.9% | 0.5% | 18.0% | 0.9% | 24.4% | 0.9% |
| Very good (%) | 30.7% | 0.5% | 23.8% | 1.0% | 24.0% | 0.8% |
| Good (%) | 28.5% | 0.5% | 31.0% | 1.2% | 27.1% | 0.9% |
| Fair (%) | 13.5% | 0.4% | 19.6% | 1.0% | 17.6% | 0.8% |
| Poor (%) | 5.5% | 0.3% | 7.5% | 0.7% | 6.8% | 0.7% |
| Death/institutionalization during year (%) | 0.8% | 0.1% | 0.6% | 0.1% | 0.5% | 0.1% |
| Priority conditions count | 2.111 | 0.030 | 2.228 | 0.057 | 1.504 | 0.049 |
| Priority for oversampling (%) | 21.3% | 0.4% | 25.8% | 1.2% | 20.3% | 1.0% |

| | Non-Hispanic White | Non-Hispanic Black | Hispanic |
|---|---|---|---|

© 2025 Schwartz AL et al. *JAMA Health Forum*.

| Insurance characteristics | Mean | Standard Error | Mean | Standard Error | Mean | Standard Error |
|---|---|---|---|---|---|---|
| Insurance source | | | | | | |
| Medicare only (%) | 16.8% | 0.6% | 14.2% | 0.8% | 8.4% | 0.7% |
| Medicare and private (%) | 19.1% | 0.5% | 10.1% | 0.9% | 3.7% | 0.4% |
| Medicare and Medicaid (%) | 3.5% | 0.2% | 9.8% | 0.9% | 9.8% | 0.9% |
| Private only (%) | 49.1% | 0.8% | 39.2% | 1.3% | 42.8% | 1.7% |
| Private and Medicaid (%) | 0.9% | 0.1% | 1.7% | 0.3% | 1.7% | 0.2% |
| Medicaid only (%) | 8.1% | 0.4% | 21.5% | 1.1% | 28.4% | 1.3% |
| Uninsured (%) | 2.4% | 0.1% | 3.5% | 0.3% | 5.3% | 0.3% |
| Private HMO (%) | 14.3% | 0.5% | 15.4% | 0.9% | 15.7% | 0.9% |
| Medicare Advantage (%) | 12.8% | 0.4% | 12.9% | 0.8% | 10.3% | 0.7% |
| Medicaid HMO (%) | 6.8% | 0.4% | 16.4% | 1.0% | 26.3% | 1.3% |

| | Non-Hispanic White | | Non-Hispanic Black | | Hispanic | |
|---|---|---|---|---|---|---|
| **Market characteristics** | Mean | Standard Error | Mean | Standard Error | Mean | Standard Error |
| Zip-code provider concentration | | | | | | |
| Physician concentration | 0.183 | 0.003 | 0.148 | 0.003 | 0.138 | 0.003 |
| Multispecialty firm concentration | 0.495 | 0.006 | 0.437 | 0.007 | 0.400 | 0.010 |
| Geographic Practice Cost Index | | | | | | |
| Work component | 1.008 | 0.001 | 1.009 | 0.001 | 1.014 | 0.001 |
| Practice expense component | 0.981 | 0.003 | 0.988 | 0.005 | 1.027 | 0.005 |
| Malpractice component | 0.916 | 0.012 | 1.033 | 0.025 | 1.040 | 0.021 |
| Region | | | | | | |
| Northeast (%) | 20.4% | 1.2% | 14.9% | 1.5% | 18.2% | 1.8% |
| Midwest (%) | 22.6% | 1.3% | 15.9% | 1.7% | 10.1% | 1.4% |
| South (%) | 39.3% | 1.6% | 64.1% | 2.2% | 42.5% | 3.0% |
| West (%) | 17.6% | 1.1% | 5.1% | 0.7% | 29.2% | 2.1% |

| | Non-Hispanic White | | Non-Hispanic Black | | Hispanic | |
|---|---|---|---|---|---|---|
| **Visit characteristics** | Mean | Standard Error | Mean | Standard Error | Mean | Standard Error |
| Clinician type | | | | | | |
| primary care physician (%) | 43.5% | 0.4% | 52.1% | 1.1% | 57.9% | 1.0% |
| specialty physician (%) | 37.9% | 0.4% | 34.0% | 1.0% | 30.3% | 0.8% |
| non-physician (%) | 18.6% | 0.4% | 13.9% | 0.9% | 11.7% | 0.7% |
| Clinician specialty | | | | | | |
| Tier 1 specialty (%) | 26.9% | 0.4% | 33.4% | 1.0% | 30.2% | 0.7% |
| Tier 2 specialty (%) | 25.5% | 0.4% | 23.3% | 0.8% | 33.1% | 1.1% |
| Tier 3 specialty (%) | 29.0% | 0.3% | 29.4% | 0.8% | 24.9% | 0.7% |
| Purpose of visit | | | | | | |
| General checkup (%) | 33.5% | 0.4% | 41.7% | 0.8% | 31.5% | 0.7% |
| Diagnosis/treatment (%) | 41.5% | 0.5% | 31.2% | 0.9% | 40.1% | 0.8% |
| Emergency (%) | 0.8% | 0.0% | 0.9% | 0.1% | 0.7% | 0.1% |
| Mental health counseling (%) | 3.4% | 0.3% | 3.0% | 0.6% | 3.0% | 0.4% |
| Follow-up/post-op (%) | 13.0% | 0.3% | 14.2% | 0.6% | 13.1% | 0.6% |
| Immunizations/shots (%) | 1.7% | 0.1% | 2.5% | 0.3% | 3.0% | 0.3% |
| Vision exam (%) | 1.0% | 0.1% | 1.2% | 0.2% | 0.9% | 0.1% |
| Pregnancy-related (%) | 0.6% | 0.1% | 0.9% | 0.2% | 1.6% | 0.2% |
| Well child exam (%) | 2.0% | 0.1% | 2.0% | 0.2% | 4.0% | 0.4% |
| Other (%) | 2.2% | 0.1% | 1.9% | 0.2% | 1.8% | 0.2% |
| Missing (%) | 0.3% | 0.0% | 0.3% | 0.1% | 0.3% | 0.1% |
| Any laboratory testing (%) | 22.3% | 0.3% | 29.0% | 0.8% | 24.7% | 0.7% |
| Any radiology testing (%) | 9.9% | 0.2% | 9.7% | 0.5% | 8.6% | 0.3% |
| Work RVUs | 3.381 | 0.013 | 3.296 | 0.028 | 3.289 | 0.026 |
| Visit has procedures without RVU but with Part B price (%) | 0.002% | 0.002% | 0.000% | 0.000% | 0.000% | 0.000% |

© 2025 Schwartz AL et al. *JAMA Health Forum*.

| | | | | | | |
|---|---|---|---|---|---|---|
| Visit has procedures without RVU or Part B price (%) | 9.15% | 0.23% | 12.58% | 0.72% | 14.92% | 0.57% |
| Part B price for non-RVU procedure codes | 0.001 | 0.001 | 0.000 | 0.000 | 0.000 | 0.000 |
| Total charge for unpriced procedures | 8.614 | 0.330 | 10.461 | 0.832 | 14.696 | 1.103 |
| Federally qualified health center (%) | 2.7% | 0.2% | 7.5% | 0.7% | 9.8% | 0.9% |

© 2025 Schwartz AL et al. *JAMA Health Forum*.

**eTable 5.** Payment Disparities for Outpatient Visits by Subgroup

| | Black-White Payment Gap | | | | Hispanic-White Payment Gap | | | |
|---|---|---|---|---|---|---|---|---|
| | % Gap | 95% CI | | | % Gap | 95% CI | | |
| **Overall** | -0.088 | -0.110 | — | -0.067 | -0.098 | -0.124 | — | -0.072 |
| | | | | | | | | |
| **Patient Characteristics** | | | | | | | | |
| Age | Chi-squared test p-value: <0.01 | | | | Chi-squared test p-value: <0.01 | | | |
| Age 65+ | -0.057 | -0.079 | — | -0.034 | -0.082 | -0.112 | — | -0.052 |
| Age 18-64 | -0.101 | -0.122 | — | -0.080 | -0.093 | -0.119 | — | -0.067 |
| Age 0-17 | -0.139 | -0.160 | — | -0.118 | -0.151 | -0.174 | — | -0.128 |
| | | | | | | | | |
| Gender | Chi-squared test p-value: <0.01 | | | | Chi-squared test p-value: <0.01 | | | |
| Male | -0.096 | -0.118 | — | -0.074 | -0.104 | -0.130 | — | -0.078 |
| Female | -0.084 | -0.105 | — | -0.062 | -0.093 | -0.120 | — | -0.067 |
| | | | | | | | | |
| Priority Conditions | Chi-squared test p-value: <0.01 | | | | Chi-squared test p-value: <0.01 | | | |
| <3 priority conditions | -0.092 | -0.113 | — | -0.071 | -0.110 | -0.134 | — | -0.085 |
| ≥3 priority conditions | -0.078 | -0.101 | — | -0.056 | -0.091 | -0.120 | — | -0.061 |
| | | | | | | | | |
| **Visit Characteristics** | | | | | | | | |
| New vs. Established | Chi-squared test p-value: <0.01 | | | | Chi-squared test p-value: <0.01 | | | |
| Initial Visit | -0.055 | -0.071 | — | -0.038 | -0.054 | -0.075 | — | -0.034 |
| Established Patient | -0.093 | -0.115 | — | -0.071 | -0.105 | -0.132 | — | -0.078 |
| | | | | | | | | |
| Provider Type | Chi-squared test p-value: <0.01 | | | | Chi-squared test p-value: <0.01 | | | |
| Specialty Physician | -0.084 | -0.104 | — | -0.063 | -0.083 | -0.109 | — | -0.058 |
| Primary Care Physicians | -0.089 | -0.110 | — | -0.067 | -0.108 | -0.134 | — | -0.082 |
| Non-Physician | -0.106 | -0.129 | — | -0.083 | -0.102 | -0.131 | — | -0.074 |
| | | | | | | | | |
| RVU | Chi-squared test p-value: <0.01 | | | | Chi-squared test p-value: <0.01 | | | |
| >median RVU | -0.064 | -0.081 | — | -0.048 | -0.070 | -0.091 | — | -0.050 |
| ≤median RVU | -0.130 | -0.160 | — | -0.100 | -0.146 | -0.183 | — | -0.109 |
| | | | | | | | | |
| **Market Characteristics** | | | | | | | | |
| Census Region | Chi-squared test p-value: <0.01 | | | | Chi-squared test p-value: <0.01 | | | |
| Northeast | -0.098 | -0.121 | — | -0.075 | -0.117 | -0.141 | — | -0.092 |
| Midwest | -0.116 | -0.136 | — | -0.095 | -0.103 | -0.126 | — | -0.079 |
| South | -0.082 | -0.104 | — | -0.061 | -0.089 | -0.118 | — | -0.059 |
| West | -0.084 | -0.105 | — | -0.063 | -0.096 | -0.119 | — | -0.074 |
| | | | | | | | | |
| Physician Concentration | Chi-squared test p-value: 0.22 | | | | Chi-squared test p-value: <0.01 | | | |
| >median MD conc | -0.092 | -0.114 | — | -0.071 | -0.083 | -0.107 | — | -0.059 |
| ≤median MD conc | -0.090 | -0.111 | — | -0.069 | -0.111 | -0.137 | — | -0.086 |
| | | | | | | | | |
| County Minority Share | Chi-squared test p-value: 0.11 | | | | Chi-squared test p-value: <0.01 | | | |
| >median share minority | -0.097 | -0.117 | — | -0.077 | -0.112 | -0.134 | — | -0.089 |
| <median share minority | -0.102 | -0.123 | — | -0.081 | -0.093 | -0.116 | — | -0.069 |
| | | | | | | | | |
| County Poverty Rate | Chi-squared test p-value: <0.01 | | | | Chi-squared test p-value: <0.01 | | | |
| >median poverty | -0.095 | -0.118 | — | -0.073 | -0.110 | -0.142 | — | -0.079 |
| <median poverty rates | -0.072 | -0.091 | — | -0.052 | -0.081 | -0.104 | — | -0.059 |

RVU = relative value units

© 2025 Schwartz AL et al. *JAMA Health Forum*.

**eTable 6.** Additional Decompositions of Payment Gaps

| Adjustments for allowable sources of payment differences | Black—White Payment Gap | | | | Hispanic—White Payment Gap | | | |
|---|---|---|---|---|---|---|---|---|
| | % Gap | 95% CI | | | % Gap | 95% CI | | |
| Unadjusted | -11.8% | -13.6% | — | -10.1% | -10.2% | -12.0% | — | -8.4% |
| Year | -11.8% | -13.6% | — | -10.1% | -10.3% | -12.2% | — | -8.5% |
| Year, visit | -11.7% | -13.4% | — | -9.9% | -11.9% | -13.7% | — | -10.1% |
| Year, visit, market | -8.8% | -11.0% | — | -6.7% | -9.8% | -12.4% | — | -7.2% |
| Year, visit, market, sociodemographic, health | -8.0% | -10.2% | — | -5.8% | -8.5% | -11.2% | — | -5.9% |
| Year, visit, market, insurance | -4.9% | -7.1% | — | -2.7% | -5.6% | -8.3% | — | -3.0% |
| Year, visit, market, insurance, sociodemographic, health | -4.1% | -6.2% | — | -1.9% | -4.3% | -6.9% | — | -1.8% |

© 2025 Schwartz AL et al. *JAMA Health Forum*.

**eTable 7.** Health Care Utilization Disparities With and Without Payment Disparities

| | Annual Utilization Outcome | | | | | | | | | |
|---|---|---|---|---|---|---|---|---|---|---|
| | Outpatient visits | | General checkups | | Pediatric outpatient visits | | Pediatric general checkups | | Pediatric mental health visits | |
| | Count | Gap as % of non-Hispanic White count | Count | Gap as % of non-Hispanic White count | Count | Gap as % of non-Hispanic White count | Count | Gap as % of non-Hispanic White count | Count | Gap as % of non-Hispanic White count |
| *With Payment Disparity* | | | | | | | | | | |
| Non-Hispanic White mean[a] | 8.26 | - | 1.93 | - | 4.81 | - | 1.33 | - | 0.89 | - |
| Non-Hispanic Black mean | 4.88 | 41% | 1.42 | 26% | 2.42 | 50% | 0.92 | 31% | 0.32 | 64% |
| Hispanic mean | 4.95 | 40% | 1.21 | 37% | 3.27 | 32% | 1.01 | 24% | 0.42 | 53% |
| *Without Payment Disparity (Model 1)* | | | | | | | | | | |
| Non-Hispanic Black mean | 5.44 | 34% | 1.59 | 18% | 2.87 | 40% | 1.09 | 18% | 0.38 | 57% |
| Hispanic mean | 5.58 | 32% | 1.37 | 29% | 3.96 | 18% | 1.22 | 8% | 0.50 | 43% |
| *Without Payment Disparity (Model 2)* | | | | | | | | | | |
| Non-Hispanic Black mean | 5.57 | 33% | 1.62 | 16% | 2.98 | 38% | 1.13 | 15% | 0.40 | 55% |
| Hispanic mean | 5.73 | 31% | 1.40 | 27% | 4.12 | 14.3% | 1.27 | 4.7% | 0.52 | 41% |

[a] Utilization metrics are adjusted for age, gender, and health characteristics listed in eTable 2. Model 1 employs price elasticity of physician supply of 1.18. Model 2 employs price elasticity of physician supply of 1.4

© 2025 Schwartz AL et al. *JAMA Health Forum*.

**eFigure.** Health Care Utilization Disparities With and Without Payment Disparities

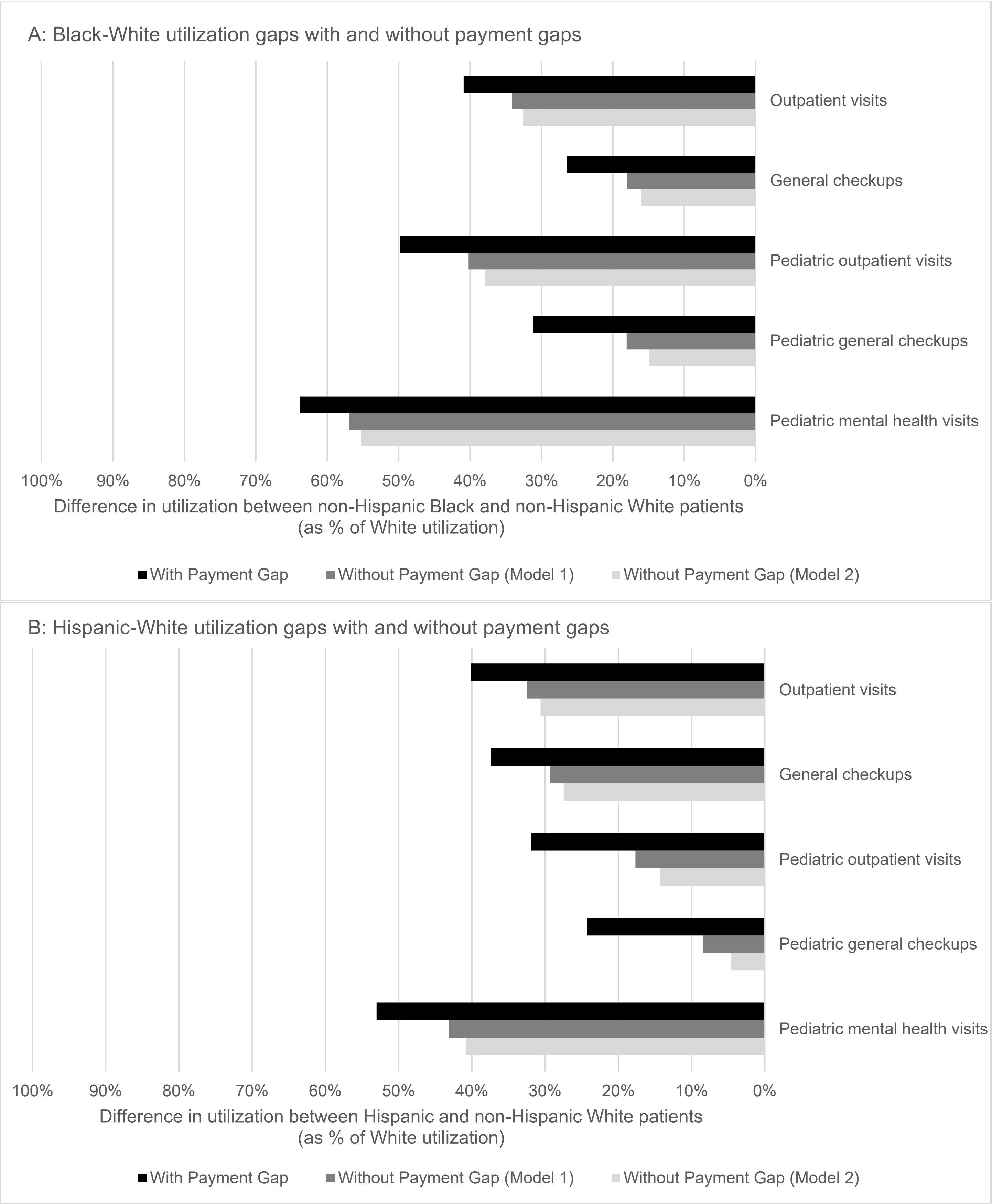


© 2025 Schwartz AL et al. *JAMA Health Forum*.
