# Supplementary material for: Payments to Physician Practices and Incentives to Serve Different Racial and Ethnic Groups
Source: JAMA Health Forum. 2025 Nov 26;6(11):e254561. doi: 10.1001/jamahealthforum.2025.4561 (PMC12658675; doi:10.1001/jamahealthforum.2025.4561)
Supplement: Supplement 2. — Data Sharing Statement [file jamahealthforum-e254561-s002.pdf]

Data Sharing Statement

Schwartz. Payments to Physician Practices and Incentives to Serve Different Racial and Ethnic Groups. *JAMA Health Forum*. Published November 26, 2025.
doi:10.1001/jamahealthforum.2025.4561

Data
Data available: No

Additional Information
Explanation for why data not available: The restricted MEPS-MPC files that are the core part of this study are not available to the public. They can only be accessed by AHRQ personnel.
